# Bromide-Mediated Silane Oxidation: A Practical Counter-Electrode Process for Nonaqueous Deep Reductive Electrosynthesis

**DOI:** 10.1021/jacsau.4c00186

**Published:** 2024-06-03

**Authors:** Mickaël E. Avanthay, Oliver H. Goodrich, David Tiemessen, Catherine M. Alder, Michael W. George, Alastair J. J. Lennox

**Affiliations:** † School of Chemistry, University of Bristol, Cantock’s Close, Bristol BS8 1TS, U.K.; ‡ School of Chemistry, University of Nottingham, University Park, Nottingham NG7 2RD, U.K.; § Modalities Platform Technologies, Molecular Modalities Discovery, GSK Medicines Research Centre, Stevenage SG1 2NY, U.K.

**Keywords:** electrosynthesis, reduction, silane, bromide, flow chemistry

## Abstract

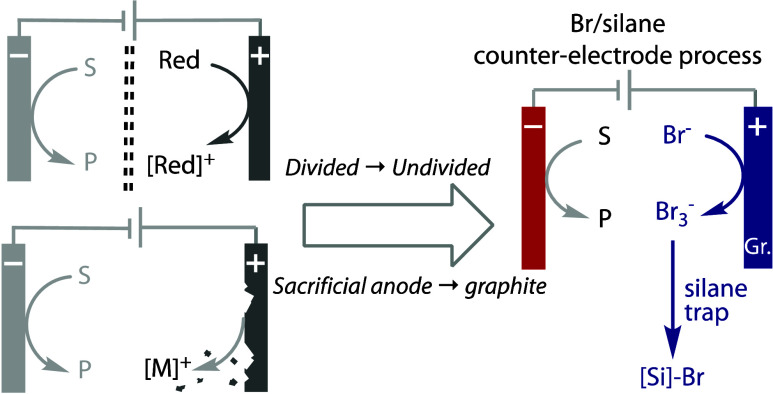

The counter-electrode process of an organic electrochemical reaction is integral for the success and sustainability of the process. Unlike for oxidation reactions, counter-electrode processes for reduction reactions remain limited, especially for deep reductions that apply very negative potentials. Herein, we report the development of a bromide-mediated silane oxidation counter-electrode process for nonaqueous electrochemical reduction reactions in undivided cells. The system is found to be suitable for replacing either sacrificial anodes or a divided cell in several reported reactions. The conditions are metal-free, use inexpensive reagents and a graphite anode, are scalable, and the byproducts are reductively stable and readily removed. We showcase the translation of a previously reported divided cell reaction to a >100 g scale in continuous flow.

Organic electrosynthesis is a rapidly evolving discipline that provides efficient, selective, safe, and environmentally friendly redox transformations to the field of organic synthesis.^1−7^ Recent developments have introduced innovative strategies to enable new and different reactivity.^8−10^ While several redox-neutral processes have been successfully realized,^11−14^ the selection of any potential renders the technique well suited to net redox transformations. While the desired redox reaction occurs on the working electrode, the circuit has to be completed via a counter-electrode process.^15^ For oxidation reactions, the most common counter-electrode process is the reduction of protons to liberate H_2_ at the cathode.^15^ The H_2_ dissipates through bubbling, thereby mitigating interference with the oxidation reaction, at least in batch mode and on regular laboratory scales.

In contrast to oxidation reactions, the counter-electrode processes available to nonaqueous reduction reactions are less general and ideal. A frequently used counter-electrode process is the oxidation of a sacrificial metal anode, such as Zn, Mg, or Al, Figure 1A-i.^16^ While practical on laboratory scales, reduction of the liberated metal ions limits the working potential that can be applied, while precipitation of insoluble metal salts can passivate the electrode and decrease its conductivity.^17−21^ There are also sustainability and scalability concerns due to the generation of stoichiometric metal waste and the requirement for specific reactor design for scale-up due to electrode degradation impacting the interelectrode gap.^22,23^ Less frequently employed options include homogeneous chemical reductants, such as amines, e.g., DIPEA, DIPA, or TEA,^24,25^ or acids, e.g., formic or pivalic acid,^10^Figure 1A-ii. The stability of the oxidation byproducts is critical in these cases as they should not compete with the cathodic reaction,^9^ and, as such, generally limits their use to milder nonaqueous reductions. For deep reductions, a divided cell can be employed, which limits interference between the oxidation byproducts and the reduction reaction, Figure 1A-iii. However, for nonaqueous electrosynthesis, divided cells are generally less practical, seldom commercially available, and increased internal resistance leads to higher applied *E*_cell_ potentials or lower currents and productivities.^26^ As ion-selective membranes are generally incompatible with organic solvents, the use of porous membranes is required, but they render the scale-up of divided cells difficult. The degradation of these separators also adds further complications and diminishes their lifetime.^22,27,28^ As a result of these challenges, the development of counter-electrode processes for nonaqueous electrochemical reduction reactions is an active field of study. For example, recent works by See and co-workers have found insightful solutions to avoid passivation of sacrificial anodes.^29^ Additionally, a system involving the use of hydrogen gas as a terminal reductant combined with quinone redox catalysis in a divided cell was elegantly demonstrated by Stahl and co-workers for large-scale nickel-catalyzed cross-electrophile coupling.^30^

**Figure 1:**
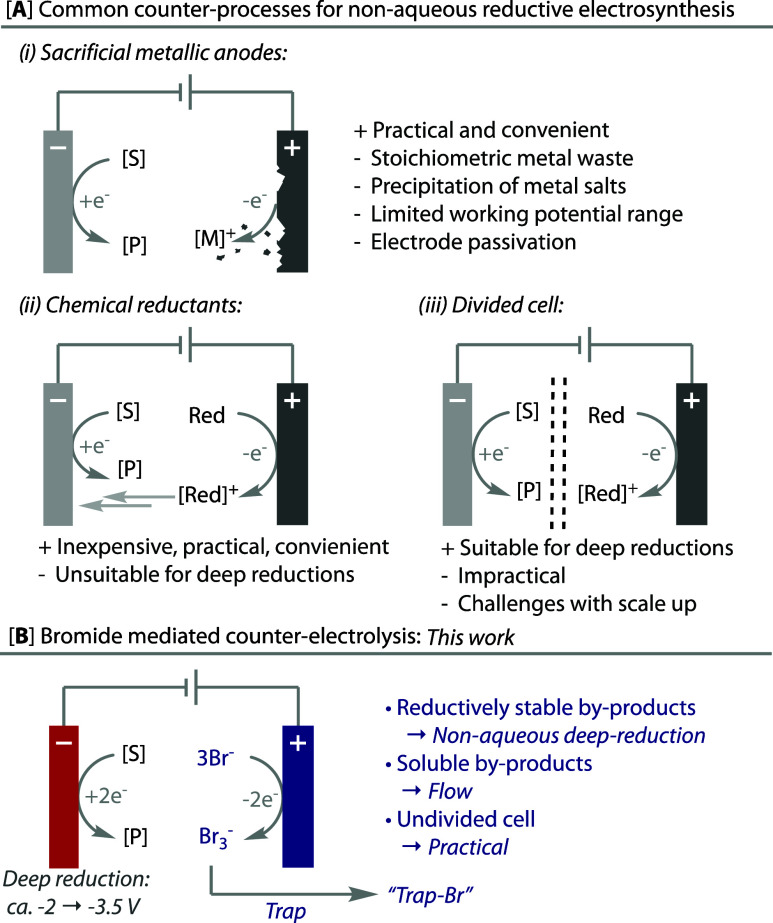
Common approaches to the counter-electrode process of nonaqueous reduction reactions, with our hypothesis for a system to address certain issues with current approaches.

We were keen to explore whether a different counter-electrode process could be developed for a scalable undivided cell that would specifically be suitable for nonaqueous deep reduction reactions; i.e., *E*_applied_ is lower than ca. – 2 V vs Fc/Fc^+^, Figure 1B. These criteria require the oxidation byproducts to be reductively stable and, for translation into scalable flow systems, also soluble. The starting point for our study was based on our previous discovery that the oxidation of bromide in divided cells serves as an efficient and inexpensive counter-electrode process for deep reduction reactions.^8,31^ As the product formed from the oxidation reaction, tribromide (Br_3_^–^), is anionic, we hypothesized that it resists migration to the cathode compartment and its subsequent reduction. Hence, our hypothesis for transitioning to an undivided cell based on bromide oxidation was to discover a trap that would transform Br_3_^–^ into a reductively stable, soluble species, Figure 1B. In this way, reductions that take place at highly reducing potentials should be achievable using simple undivided cell setups, such as beakers or Schlenk tubes, as well as on larger scales and in flow.

The electrochemical hydrodefluorination of trifluoromethylarenes requires very deep potentials in the range of −2.5 to −3.5 V (vs Fc/Fc^+^) and hence served as a suitable test reaction. The reaction is reported using TMSCl (6 equiv) as a fluoride trap in a divided cell with the anodic oxidation of Br^–^ as the counter-electrode process,^8^ entry 1, Table 1. Directly transferring these conditions to an undivided cell using an inexpensive graphite rod anode did not afford the desired product **2a** in good yield, entry 2, as would be expected from cycling of the bromide/tribromide redox couple. A range of additives that have been reported as chemical reductants in electrosynthetic reductions were tested (entries 3–5) in place of the bromide oxidation but did not lead to any observed product **2a**. Hence, in the presence of bromide, we screened a variety of coreductants to scavenge the tribromide, including phosphine, amine, and silane additives (entries 6–11). The best results came from using triethylsilane (TES, entry 11), which, after some adjustment of the loadings, electrochemical parameters, and the stirring rate, led to an optimized yield of hydrodefluorinated product **2a** (entry 12). Without bromide or co-reductant, the yield dropped significantly (entries 13–14).

**Table 1:**
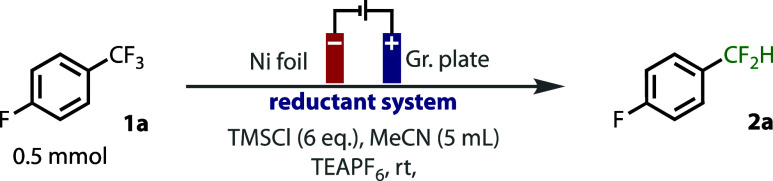
Optimization and Benchmarking of Hydrodefluorination in an Undivided Cell^a^

entry	reductant system^b^	NBu_4_Br (equiv)	yield/%
1	divided cell (lit):^8^ 5 mA	3	76
2	undivided cell, 5 mA	3	24
3	*i*-Pr_2_NEt (4)	n/a	0
4	PivOH (3)	n/a	0
5	thiourea (1)	n/a	0
6	PPh_3_ (3)	3	7
7	NPh_3_ (3)	3	28
8	DPS (3)	3	32
9	TMDS (3)	3	41
10	TEOS (3)	3	18
11	Et_3_SiH (3)	3	42
**12**	**Et_3_SiH (3), 20 mA, 0.2 M, 4*F***	**1.5**	**69**
13	Et_3_SiH (8)	0	36
14	none	0	27

a ^19^F NMR yields.

b Unless otherwise stated, 0.1 M **1a**, run at 50 mA for 3F. Values in parentheses of reductants refer to the number of equivalents used. TES: triethylsilane, DPS = diphenylsilane, TMDS = 1,1,3,3-tetramethyldisiloxane, TEOS = triethoxysilane.

The yield from our optimized conditions (entry 12) compares closely to that originally reported using a divided cell (entry 1), although a marginally increased level of over-reduction was observed (see Supporting Information (SI)). An added advantage of switching to an undivided cell is a lower overall cell resistance, which facilitates the application of a much higher current. Hence, we were able to increase the current 4-fold compared to the divided cell conditions (20 vs 5 mA) and reduce the reaction time. The loading of reagents used equates to 1 equiv of Et_3_SiH and 0.5 equiv of NBu_4_Br per F, which corresponds to a net process where each equivalent of Et_3_SiH provides one equivalent of electrons, consuming half an equivalent of Br^•^ mediator in the process to form the reductively stable Et_3_Si–Br.

CV studies indicate that Et_3_SiH is not readily oxidized compared to the mild oxidation of bromide, Figure 2A. However, the presence of Et_3_SiH increases the current response of the second oxidation wave of NBu_4_Br while suppressing the reduction wave. This, and the observation of the discoloration of a solution of Bu_4_NBr_3_ with Et_3_SiH, support our hypothesis that silane quenches the tribromide and bromine formed on the anode.^32^ The level of current in the second oxidation feature is dramatically enhanced, demonstrating that bromine is quenched faster than tribromide. This process liberated HBr and Et_3_SiBr. Evidence, albeit indirect, for the formation of Et_3_SiBr was gained from control reactions (see SI for details): while Et_3_SiF could not be observed between Et_3_SiH and NBu_4_F, it cleanly formed if Et_3_SiH was prestirred with Br_3_^–^before addition of NBu_4_F. This observation can be rationalized through the formation of intermediate Et_3_SiBr, which, in the absence of fluoride, likely rapidly hydrolyzes to TESOH or TES_2_O. Cathodic proton reduction from dissociated HBr, Figure 2B, can also account for the lower faradaic efficiency of this deep reduction compared to the divided cell conditions.^8^

**Figure 2:**
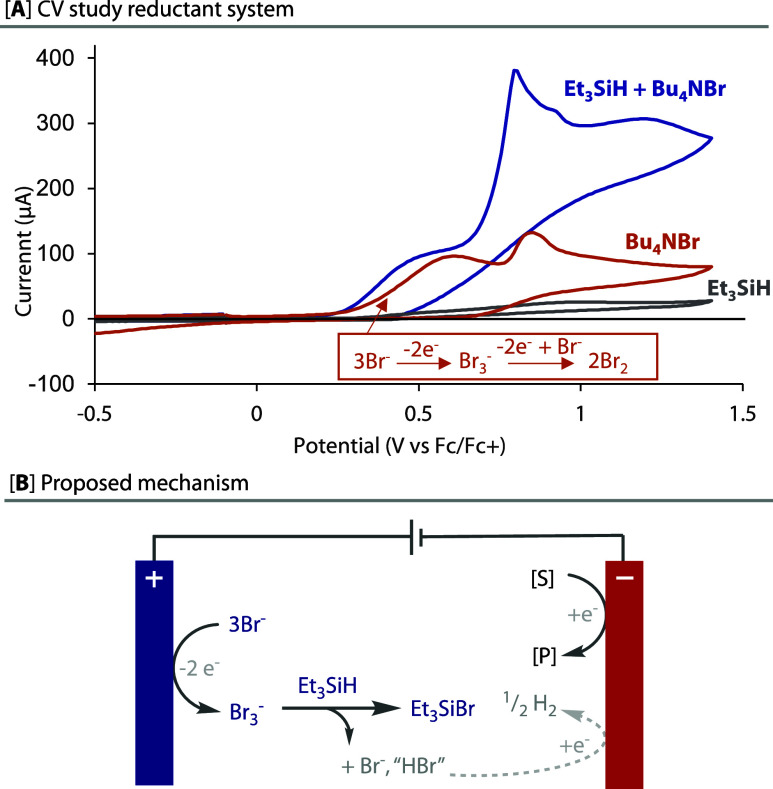
Mechanistic insight and proposal.

To gauge how our system would tolerate oxidatively sensitive functional groups, we conducted a robustness screen in which we subjected various functional groups to NBu_4_Br_3_ and then monitored how the presence of Et_3_SiH affects this reactivity. We found that the presence of Et_3_SiH effectively protected functional groups from Br_3_^–^ that were otherwise readily brominated, such as electron-rich arenes, styrenes, and triaryl phosphines (see SI for details). These control experiments demonstrate successful quenching of the anodically formed oxidant and provide confidence that electron-rich substrates should not be affected by the bromide/silane system.

We applied this undivided, metal-free, counter-electrode process to a variety of other substrates and also to other reactions that have been reported either in a divided cell or with a sacrificial metal anode, Figure 3. To assess our conditions most fairly against those reported, we elected to keep the same solvents, reactant equivalents, scales, concentrations, and cathode materials to that originally reported and only vary the equivalents of TES, NBu_4_Br and electrochemical parameters. Due to their very low polarity, the removal of the terminal silylated byproducts, i.e., mostly TES_2_O or unreacted reagents, is readily achievable via silica gel chromatography, and hence, isolation and purification were straightforward in the majority of cases. Our reductant system was amenable to all the transformations that we tested, with the exception of only an electrochemical Birch reduction^18^ and a cross-electrophile coupling.^19^

**Figure 3:**
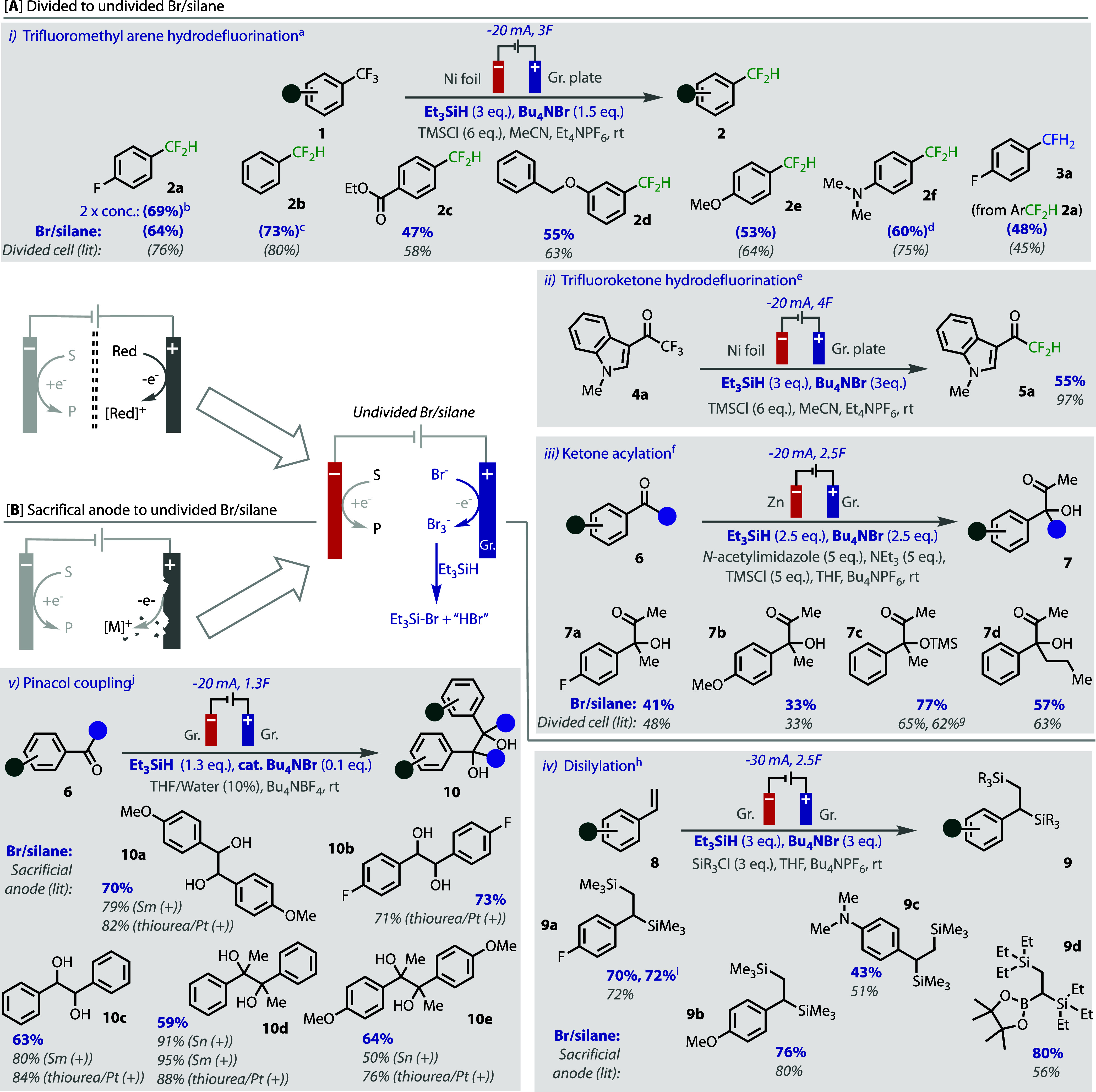
“Replace and Reduce”. Isolated yields are shown, and NMR yields are shown in parentheses. Conditions shown in blue are associated with the counter-electrode system, and those shown in gray are unchanged from the literature. ^a^Literature divided cell conditions from ref (8); ^b^run at 0.2 M, as opposed to 0.1 M and only 3 equiv. TMSCl; ^c^run for 4F; ^d^run for 6F; ^e^Literature divided cell conditions from ref (31); ^f^Literature divided cell conditions from ref (33); ^g^run in our hands, an average of 2 runs; ^h^ Literature sacrificial anode conditions from ref (34); ^i^Run in the IKA ElectraSyn using TBAClO_4_ instead of TBAPF_6_; ^j^Literature conditions from refs (35−37).

More electronically diverse substrates in the arene hydrodefluorination reaction were first tested with the new undivided conditions, as shown in Figure 3Ai. In keeping with the electronic ambivalence of the original divided cell conditions,^8^ good yields that compared well with that reported were observed for both electron-poor substrates and, most impressively, difficult-to-reduce electron-rich substrates. Oxidatively sensitive functional groups were also tolerated, e.g., dimethylaniline **2f**. These two observations highlight how the conditions are suitable both for deep reduction reactions and that the oxidation byproducts of the counter-electrode process are sufficiently quenched so as not to react undesirably.

The second hydrodefluorination of difluoromethyl arene **2a** to the monofluoromethyl arene **3a** occurs at deeper reduction potentials,^8^ but worked well nonetheless, Figure 3A-i. The hydrodefluorination of trifluoromethylketones (**4**) has previously only been achieved using electrochemical reduction in a divided cell,^31^ however, a good yield of product **5b** was achieved using our undivided bromide/silane system, Figure 3A-ii.

The electrochemical acetylation of ketones has also only previously been reported in a divided cell.^33^ In the reported procedure, different solvents were employed for each electrode compartment and the anolyte was contained in a ceramic diaphragm; hence, we deemed an undivided adaptation of the setup would be advantageous. Pleasingly, the undivided bromide/silane system successfully afforded several products (**7**) in moderate to good yields, which all compared well to the reported conditions,^33^Figure 3A-iii. Of note is the successful generation of product **7c**, which is acid-sensitive. This result demonstrates that the conditions are not overly acidic despite the formal generation of protons from the counter-electrode process.

We next endeavored to explore the bromide/silane system in reactions originally reported with sacrificial metal electrodes. Lin and co-workers reported the disilylation of styrenes using a sacrificial Mg anode as the electron source.^34^ We reasoned it would be advantageous to replace the Mg anode because insoluble microparticles formed, and plating of the cathode was noted that could lead to a hazard if the reaction was run with wet solvent or alcoholic substrates. Using the bromide/silane counter-electrode system on a graphite anode afforded good to very good yields of product **9** that compared well to those reported, including very electron-rich substrates, e.g., **9d** and **9e**, that would be liable to bromination in the absence of a suitable trap. The same commercially available equipment could also be successfully used. For the trimethylsilylation (**9a**–**c**), selectivity was retained, with no triethylsilane observed in the products. Removal of silane-related byproducts was possible using an adapted procedure involving ethanolamine addition and purification using chromatography, see SI.

The bromide/silane system was tested under aqueous conditions using an electrochemical reductive aldehyde pinacol coupling. This reaction has potential broad utility in the diversification of biomass feedstocks^36,38−40^ and has been reported with the use of a sacrificial Sn^35^ or Sm^36^ electrode or using thiourea on platinum electrodes.^37^ Our undivided conditions represent the only example of a metal-free pinacol coupling of benzaldehydes. Pleasingly, with very little optimization, the bromide/silane system on a graphite electrode led to good to very good isolated yields of diols **10**. Under these aqueous conditions, catalytic and substoichiometric quantities of bromide could be used, with as little as 10 mol % effecting the desired transformation. This should be due to the liberation of bromide ions following the hydrolysis of Et_3_SiBr, an effect that can be observed by CV (see SI for details). An enhanced Faradaic efficiency is also observed, which should be due to the milder applied reduction potential not reducing the liberated protons.

In order to test the utility of these conditions toward scale-up, we investigated the trifluoromethyl arene hydrodefluorination in continuous flow, in which the reported conditions had demonstrated a scale of up to 5 mmol and a productivity of 0.2 g·day^–1^.^8^ For these investigations, we used an electrochemical Taylor vortex reactor (ElectroVortex),^41,42^ which is similar to that previously reported for the scale-up of continuous photochemistry,^43,44^ and which we have previously used for electrochemical benzylic amidation.^45^ Because it is so readily available and inexpensive in large quantities, benzotrifluoride **1b** served as an optimization substrate, Figure 4A. The reaction of **1b** to **2b** was tested at several flow rates, electrode spinning frequencies, and currents. Increasing the rotation speed of the electrode afforded higher yields of **2b** while decreasing the overall conversion due to better mixing, leading to lower over-reduction. Unlike in the batch-divided system,^8^ it was found the reaction could be performed without any precaution to exclude moisture or air, which significantly simplifies the process, and an inexpensive stainless-steel cathode could be used. We found the best yield (^19^F NMR) was found using rotation speeds of 2000 rpm, a charge of 3.8F, and a flow rate of 5 mL·min^–1^, leading to a peak projected productivity of 143 g·day^–1^ entry 4 Figure 4A. We tested these conditions over a period of 4 h and observed no significant change in conversion or product distribution (see SI for details).

**Figure 4:**
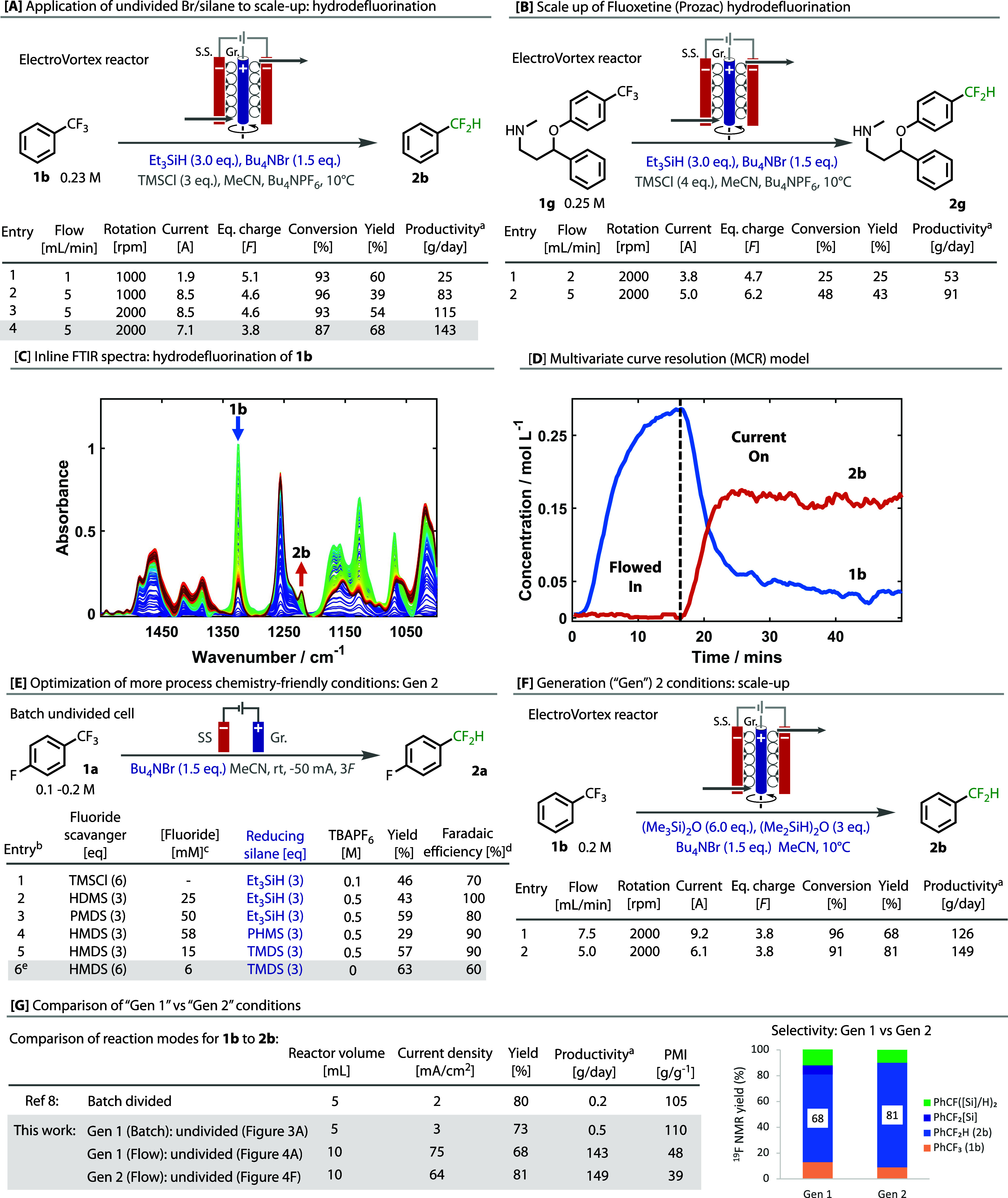
Scale-up and analysis of hydrodefluorination in flow, with the development of new “generation 2 conditions”. Yields were calculated using quantitative ^19^F NMR with fluorobenzene as the internal standard. ^a^Extrapolated from steady-state productivity; ^b^Entries 1–5 1b = 0.1 M, entry 6:1b = 0.2 M. ^c^Calculated by measuring the quantity of TIPSF detected (quant. ^19^F NMR) after the addition of TIPSCl (1 equiv) to the reaction after electrolysis; ^d^FE = (*F*_passed_)/(2 × yield_CF2X_ + 4 × yield_CFX2_ + 6 × yield_CX3_); ^e^0.2 M **1a** used, at −25 mA; ^f^Reaction performed on **1a**.

A scale-up for the hydrodefluorination of the antidepressant drug fluoxetine (Proxac) **1g** was attempted in flow. With very limited optimization, a good yield of **2g** could be afforded after passing 6.2F at a flow rate of 2 mL·min^–1^, which extrapolates to a projected productivity of 91 g·day^–1^. This experiment demonstrates the possibility of using these conditions to perform late-stage functionalization of drugs on practical scales, Figure 4B.

Performing the reaction in flow also enabled the transformation to be monitored using inline Fourier transform infrared (FTIR), Figure 4C. Clear consumption of **1b** was observed by a large reduction in the C–F stretch at 1325 cm^–1^, with the corresponding formation of **2b** being observed at 1200 cm^–1^. A multivariate curve resolution (MCR) model, as we have used previously,^45^ was developed, Figure 4D, which shows predicted concentrations and gives a visual representation of relative reaction stability.

Although these conditions proved scalable in flow, we were conscious that further improvements could be made, specifically to the hydrodefluorination, in relation to the process chemistry friendliness and sustainability of the reaction. For example, TMSCl is water-sensitive and can release HCl, cheaper reducing silanes than TES are available,^46^ and supporting electrolyte salt (TBAPF_6_) is used. Hence, we sought to develop a second, “Generation 2”, set of conditions, as shown in Figure 4E.

To replace the fluoride scavenger, TMSCl, we surveyed a range of replacements, including ^*i*^PrOTMS, TMS_2_O hexamethyldisilane (HMDS), and polymeric poly(dimethylsiloxane) (PDMS), see SI for full details. While each of them worked to give good yields of **2b**, HMDS was deemed the most suitable replacement, as it is very inexpensive, and led to the best fluoride scavenging and faradaic efficiency **2a** (entry 3 vs 2 Figure 4E, see SI for full details) and was more practical to use than the viscous PDMS. To replace Et_3_SiH, we tested the inexpensive (Me_2_SiH)_2_O (TMDS) and the polymeric [(MeSiH)O]*_n_* (PHMS). Similar to PDMS, PHMS was viscous and poorly soluble in MeCN, leading to a poor reaction profile (entry 4). However, TMDS outperformed Et_3_SiH and led to the formation of **2a** in good yield (entry 5). With the use of TMDS and TMS_2_O, as replacements for TMSCl and Et_3_SiH, we observed less over-reduction and no silylated side products. Finally, we investigated if the supporting electrolyte, NBu_4_PF_6_, could be removed, as the use of NBu_4_Br alone could provide sufficient conductivity to the medium. When removing the supporting electrolyte, we were able to maintain a good yield of the product after increasing the concentration and the charge passed (entry 6).

The newly developed generation 2 conditions were then explored in a continuous flow by using the ElectroVortex. Pleasingly, these conditions outperformed the Gen 1 conditions, Figure 4A at the same equivalent charge of 3.8F, affording **2b** with a yield of 81% and a projected productivity of 149 g·day^–1^, Figure 4F. This yield was in fact superior to the original yield reported in a divided cell (80% ^19^F NMR).^8^

While the reported productivity in the divided cell was low (entry 1, Figure 4G), a small improvement was made initially with the new bromide/silane counter-electrode system in batch (entry 2), but a substantial improvement on moving to flow (entries 3 and 4). This great improvement in productivity is due to the excellent mixing created in the Vortex reactor and the substantially higher currents that are applied to the reaction solution (cf. 75 vs 3 mA·cm^–2^). The Process Mass Intensity (PMI) was much improved going from batch to flow, Figure 4G, and the Gen 2 conditions indeed proved better than the Gen 1 conditions in flow. Importantly, the transition to this reactor was enabled by the use of our undivided bromide/silane counter-electrode system.

In summary, we have disclosed a new counter-electrode process for nonaqueous electrochemical reduction reactions, in which bromide and triethylsilane work in concert to provide the reducing equivalents. We have demonstrated its applicability toward several transformations that were previously reported either in divided cells or with sacrificial anodes, including highly inert deep reductions, with good tolerance to challenging electron-rich substrates. A key feature that contributes to the success of this system is that the reagents and byproducts are reductively stable, which enables a wide usable potential window. Translation of the deeply reductive hydrodefluorination from a divided cell into flow using the ElectroVortex reactor demonstrates that the system is amenable to larger scales. A more process chemistry-friendly set of generation 2 conditions was developed that facilitated a cheaper and more efficient reaction with higher yield and productivity up to 150 g·day^–1^. While this system will not be suitable for all nonaqueous reactions and conditions, we expect it to become part of the suite of counter-electrode systems that will be tested in the development of new electrochemical reduction reactions, and therefore, its use will broaden well beyond that demonstrated herein.

## Methods

Trifluoromethylarene hydrodefluorination: Et_4_NPF_6_ (1.5 mmol, 3.0 equiv), Bu_4_NBr (0.75 mmol, 1.5 equiv) and the trifluoromethylarene substrate (0.50 mmol, 1.00 equiv) were combined with MeCN (5 mL), Et_3_SiH (1.5 mmol, 3 equiv) and TMSCl (3.0 mmol, 6.0 equiv) under an inert atmosphere. Electrolysis was performed with Ni/graphite electrodes at 20 mA for 7200 s (3F).

Acetophenone acetylation: *N*-acetylimidazole (2.50 mmol, 5.0 equiv), Bu_4_NPF_6_ (0.25 mmol, 0.3 M), Bu_4_NBr (1.25 mmol, 2.5 equiv) and the substrate (0.500 mmol, 1.00 equiv) were combined with anhydrous tetrahydrofuran (THF) (7.5 mL), Et_3_N (2.50 mmol, 5.0 equiv) TMSCl (2.50 mmol, 5.0 equiv) and Et_3_SiH (1.25 mmol, 2.5 equiv) under an inert atmosphere. Electrolysis was performed with Zn/graphite electrodes at 20 mA for 6000 s (2.5F).

Alkene disilylation: Bu_4_NPF_6_ (1.8 mmol, 1.8 equiv) and Bu_4_NBr (3.0 mmol, 3.0 equiv) were combined with anhydrous THF (9 mL), the substrate (1.00 mmol, 1.00 equiv), Et_3_SiH (3.0 mmol, 3.0 equiv) and the trialkylsilane (3.0 mmol, 3.0 equiv). Electrolysis was performed with graphite/graphite electrodes at 30 mA for 8000 s (2.5F).

Metal-free pinacol coupling: Bu_4_NBF_4_ (1.0 mmol, 1.0 equiv) Bu_4_NBr (0.1 mmol, 0.1 equiv) and the substrate (1.00 mmol, 1.00 equiv) were combined with THF (4.5 mL), water (0.5 mL) and Et_3_SiH (1.3 mmol, 1.3 equiv). Electrolysis was performed with graphite/graphite electrodes at 20 mA for 6240 s (1.3F).

## Data Availability

The data underlying this study are available in the published article and its Supporting Information.

## Supporting Information Available

Experimental and computational procedures; characterization data for known and new compounds and spectra for new compounds (PDF)
