# Supplementary material for: Bromide-Mediated Silane Oxidation: A Practical Counter-Electrode Process for Nonaqueous Deep Reductive Electrosynthesis
Source: JACS Au. 2024 Jun 3;4(6):2220–7. doi: 10.1021/jacsau.4c00186 (PMC11200245; doi:10.1021/jacsau.4c00186)
Supplement: Supplementary file 1 — au4c00186_si_001.pdf [file au4c00186_si_001.pdf]

Supporting Information

Bromide-Mediated Silane Oxidation: A Practical Counter-Electrode Process for Non-Aqueous Deep Reductive Electrosynthesis

Mickaël E. Avanthay,[a] Oliver H. Goodrich,[b] David Tiemessen,[b] Catherine M. Alder,[c] Michael W., George,[b] and Alastair J. J. Lennox*[a]

[a] School of Chemistry, University of Bristol, Cantock's Close, Bristol, BS8 1TS (UK) *a.lennox@bristol.ac.uk

[b] School of Chemistry, University of Nottingham, University Park, Nottingham, NG7 2RD, (UK)

[c] Modalities Platform Technologies, Molecular Modalities Discovery, GSK Medicines Research Centre, Stevenage, SG1 2NY, (UK)

Contents

General Experimental Details 3

Batch electrochemical setups 4

Flow electrolysis setup 6

Optimisation table: 7

Mechanistic experiments 11

Oxidative stability to Br_3^-/TES system: 18

Undivided trifluoromethylarenes hydrodefluorination (General procedure 1) 21

Undivided trifluoromethylketone hydrodefluorination 25

Undivided acetophenones acetylation (General procedure 2) 26

Metal-free bissilylation of styrenes 29

Metal-free disilylation of styrenes in a Schlenk tube (General procedure 3) 29

Metal-free disilylation of 4-fluorostyrene using the ElectraSyn 31

Metal-free pinacol coupling (General procedure 4) 32

Unsuccessful reactions 36

Flow electrochemical experiments using the Electrovortex Reactor 38

Optimisation of the electrochemical synthesis of (difluoromethyl)benzene, “Generation 1 conditions” 39

Multigram synthesis of (difluoromethyl)benzene in continuous flow, “Generation 1 conditions” . 40

Electrochemical synthesis of (difluoromethyl)benzene, using “Generation 2 conditions” 42

References 45

General Experimental Details

Techniques

Manipulations involving air and moisture-sensitive materials were conducted employing standard Schlenk-line and glovebox techniques, using vacuum lines attached to a double manifold with greaseless J. Youngs valves equipped with an oil pump (0.1 mmHg) under an atmosphere of dry nitrogen. All glassware was dried overnight before use, in a 180 ^{o}C oven and then allowed to cool under vacuum at 0.05 mbar. The removal of solvents in vacuo was achieved using a Büchi rotary evaporator (bath temperatures up to 40 °C) at a pressure of 15 mmHg (diaphragm pump), or at 0.05 mbar (oil pump) on a vacuum line at room temperature. The addition of < 200 µL of liquids was *via* a Gilson PIPETMAN p20, for larger volumes standard syringe practices were employed.

Solvents

Anhydrous MeCN was dried using an Anhydrous Engineering alumina column drying system situated in the University of Bristol's chemistry department. It was collected using a Strauss flasks using a gastight J. Youngs valve then degassed by freeze-pump-thaw. Deuterated solvents for NMR analysis were purchased from Sigma Aldrich. Anhydrous THF was purchased from Across Organics (anhydrous, stabiliser-free). All other solvents, including non-anhydrous THF and MeCN were purchased lab grade and used without any purification.

Chromatography

TLC analysis was performed on Merck Silica gel 60F254 glass-backed plates. Visualisation was achieved by UV fluorescence (254 nm) or staining with basic KMnO4 or PMA.
Flash column chromatography was conducted using Merck 60 silica: 230-400 mesh (40-63 µm) or using an automated flash purification system(Biotage Selekt or Buchi Pure C-850 Flashprep) using Biotage Sfar HC Duo pre-packed columns of size 5 g, 25 g or 50 g..
Preparative Reverse-Phase HPLC was performed on a Buchi Pure C-850 FlashPrep using a Buchi PrepPure column (250 x 10 mm, C18, spherical 5 µm particle size, 100 Å pore size) and a 5 mL sample loop. The following method was used:
Phase A: 5% MeCN + 0.1 % formic acid in water; phase B: 0.1% formic acid in MeCN; flow rate: 7 mL/min.
10 min equilibration at 20% B, then injection in 0.5 mL DMSO, then 3 min elution at 20% B, then 10 min gradient 20-60% B.

Reagents

All reagents were purchased from TCI UK, Apollo Scientific, Sigma Aldrich, Alfa Aesar or Fluorochem and used as received unless otherwise stated. Et_4NPF_6 and Bu_4NClO_4 were purchased from Sigma Aldrich and stored under a dry atmosphere (in a desiccator over self-indicating silica gel or in a glovebox, respectively) between uses. Bu_4NPF_6 and Bu_4NBr were recrystalised (from EtOH and from EtOAc, respectively) and dried under vacuum at 80°C overnight before use, and stored in a desiccator over self-indicating silica gel between uses.

Analysis

NMR spectra were recorded on Bruker Nano 400 or Bruker Advance III HD 500 cryo spectrometers. Chemical shifts (δ) are quoted in parts per million (ppm), referenced to the residual solvent peak (1H and 13C NMR) and coupling constants (J) are given in Hz. Multiplicities are abbreviated as: s (singlet), d (doublet), t (triplet), q (quartet), m (multiplet) or combinations thereof. NMR shifts for novel compounds have been assigned with the use of the appropriate 2D NMR experiments, such as COSY, HSQC and HMBC. Infrared spectra were recorded using a Perkin Elmer Spectrum Two FTIR spectrometer.

Electrochemical analytical techniques

All cyclic voltammetric (CV) experiments were performed at room temperature using a MultiPalmsens 4. CV experiments were carried out with a working electrode (GC = glassy carbon, Pt, Au, Ni = 1-3 mm diameter), a counter electrode (platinum wire) and a 0.01 M Ag/$AgNO_3$ reference electrode. All working electrodes were polished before each experiment. Before each CV, the solution was stirred for approximately 10 seconds, whilst being degassed by a stream of N_2. The CV cell was maintained under an atmosphere of N_2 during analysis. After analysis, ferrocene was added to reference the data.

Batch electrochemical setups

Electrode materials and suppliers

Zinc plates, magnesium plates and graphite plates (8 x 52.5 x 2 mm) were purchased from IKA.

Pt wire was purchased from Advent research materials: diameter 0.4 mm, 99.99% temper annealed, part number PT5441. Platinum electrodes were made as described in our previous report[1] by wrapping platinum wire around PTFE tubing to create a surface area approximately ~1 cm^2. The platinum wire was then fed through PTFE tubing by creating a small hole on the side of the tubing. The wire was then spot-welded to a copper wire.

Stainless Steel grating was purchased from GERAWOO via Amazon: 304 Stainless Steel Mesh Sheet,20 Mesh.

Ni foil was purchased from Alfa Aesar: 0.05 mm (0.002”) thick, annealed, 99+% (metals basis), part number 42634.CH and cut to size with scissors.

Aluminium rods were purchased from Alfa Aesar: 6.35 mm (0.25”) dia, 99.95% (Metals basis), part number 044850.G1 and machined to size (6 cm length) in-house.

Electrode cleaning

Ni foil was discarded after each use. Other electrodes were sonicated in MeOH or MeCN then rinsed with acetone. Zn, Gr, Mg, and Al electrodes were sanded before us. Zn, Mg, and Al were scoured with 20% aq. HCl between polishing if polishing did not afford a smooth finish.

Electrochemical equipment

Schlenk tube

Electrode wire were fed through a 3D printed polypropylene stopper, attaching to the electrode material either directly or via a crocodile clip (purchased Amazon, sold for spray-painting or DIY decorative arts). Blue tack was used to ensure airtightness around the wires or crocodile clips.

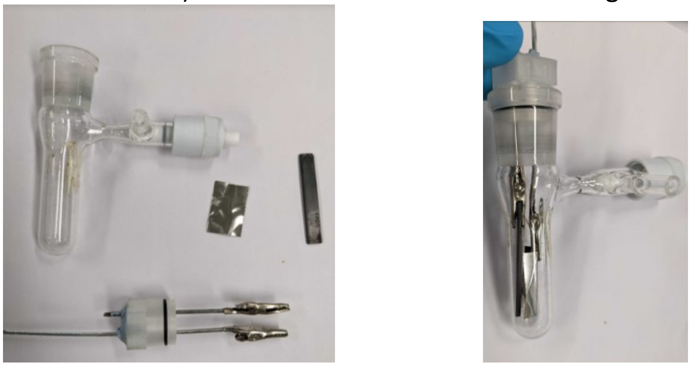

Figure S1: Example electrochemical setup with crocodile clips/polypropylene lid, Ni foil and Gr. Plate.

ElectraSyn vials
Electrasyn vial and lids were purchased from IKA and run on either an ElectraSyn 2.0, a MultiPalmsens 4 or a PLH250 Aim-tti PSU.

Flow electrolysis setup

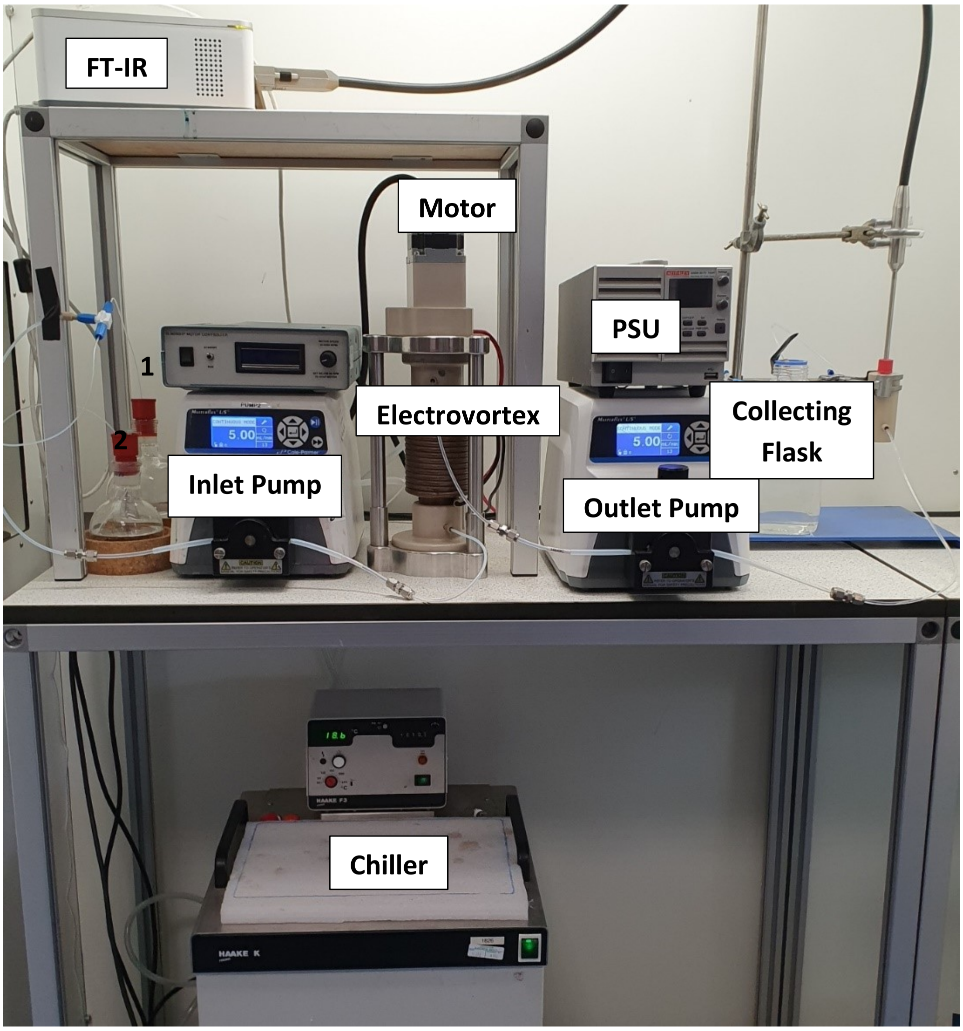

Figure S2: Annotated electrovortex setup.

The electrovortex has a 1.25 mm gap size and a reaction volume of 10.8 mL, using a graphite rod (inner electrode) with a stainless-steel outer (outer electrode) and the electrodes are attached to a 720 w power supply unit (PSU). Rotation of the graphite rotor is supplied by a custom-built motor controller attached to a brushless motor. Both the inlet and outlet pumps are peristaltic pumps using PTFE pump heads with 1/8th OD PFA tubing. The chiller uses a mixture of IPA and H_2O (1:1).

Online analysis is obtained using a FT-IR attached with a 6.3 mm AgX DiComp probe inserted into a PEEK IR cell, spectra were collected in 16 scan averages every 15 seconds.

Optimisation table
Optimisation of the undivided hydrodefluorination

Preliminary studies were performed using a Ni gauze cathode, in which many different co-reductants, solvents, and electrochemical parameters were tested. However, these studies did not lead to easily interpreted results, possibly due to the difficulty in cleaning of the gauze. Ni foil gave results that were more easily interpreted and therefore we show these.

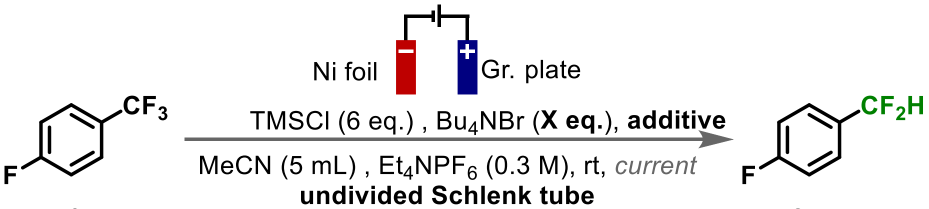

1a
0.5 mmol

2a

Table S1: Optimisation of the undivided hydrodefluorination of trifluoromethylarenes. ^{19}F NMR yields relative to internal C_6F_6 standard.[b]Pt wire as anode [a] Reaction run at 0.2 M, as opposed to 0.1 M and only 3 eq. TMSCl.

Entry	Bu_4NBr eq.	Additive	Electricity	Conversion [%]	Yield **2a** [%]	Yield "CF_1" [%]
1	0	*i*-Pr_2NEt 4 eq.	50 mA, 3*F*	0	0	0
2	0	PivOH 3 eq.	50 mA, 3*F*	0	0	0
3	0	Thiourea 1 eq.	50 mA, 3*F*	0	0	0
4[a]	0	Thiourea 1 eq.	50 mA, 3*F*	0	0	0
5	3	None	50 mA, 3*F*	26	24	0
6	3	Et_3SiH 3 eq.	50 mA, 3*F*	85	42	26
7	3	$(Me_2SiH)_2O$ 3 eq.	50 mA, 3*F*	70	41	9
8	3	Ph_2SiH_2 3 eq.	50 mA, 3*F*	54	32	5
9	3	$(EtO)_3SiH$ 3 eq.	50 mA, 3*F*	25	18	0
10	3	PPh_3 3 eq.	50 mA, 3*F*	51	7	0
11	3	NPh_3 3 eq.	50 mA, 3*F*	36	28	0
12	3	Et_3SiH 8 eq.	50 mA, 3*F*	85	42	0
13	4	Et_3SiH 8 eq.	50 mA, 3*F*	77	48	0
14	0	Et_3SiH 8 eq.	50 mA, 3*F*	34	25	0
15	0.5	Et_3SiH 8 eq.	50 mA, 3*F*	41	33	0
16	1.2	Et_3SiH 8 eq.	50 mA, 3*F*	85	51	0
17	4	Et_3SiH 8 eq.	20 mA, 3F	78	63	13
18	1.5	Et_3SiH 3 eq.	20 mA, 3F	72	64	6
19[a]	1.5	Et_3SiH 3 eq.	20 mA, 4F	90	69	18

Impact of the charge on the product distribution and selectivity:

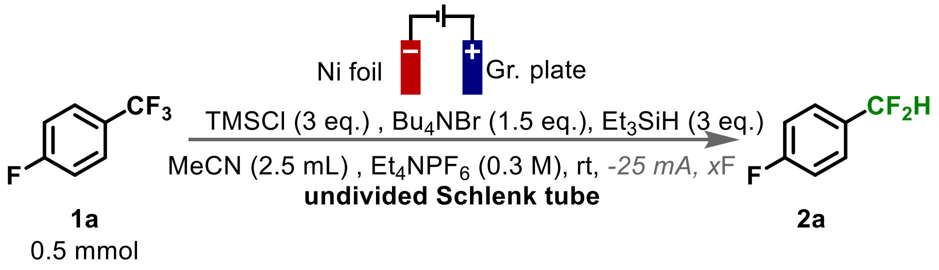

The entry 15 of Table S1 was run for two different amounts of charge in order to assess the selectivity against charge passed (Table S2). Increasing the charge from 3*F* to 4*F* led to a 29% point increase in conversion leads only to 13% point increase in "CF_2" species. This can be explained by an increase of overreduction of the "CF_2" species to "CF_1" species by 13% point. As a result, the choice of the amount charge to pass is a balancing act between increasing conversion and limiting over-reduction.

Table S2: Impact of the charge in the double concentration experiment ^{19}F NMR yields relative to internal C_6F_6 standard. "CF_2" refers to all monodefluorinated species detected, "CF_1" refers to all didefluorinated species detected.

Entry	Charge	Conversion [%]	$ArCF_2H$ (**2a**) [%]	$ArCF_2TMS$ [%]	$ArCF_2TES$ [%]	$ArCFH_2$ (**3a**) [%]	ArCFHSi [%]	*Total "CF_2"* [%]	*Total "CF_1"* [%]	*Selectivity (CF_2H : CFH_2)*
1	3*F*	61	57	4	0.7	4	0.2	*61*	*5*	*14:1*
2	4*F*	90	69	5	0.5	13	4	*74*	*18*	*5:1*

The selectivity at 3*F* is 14:1, while at 4*F* the selectivity has been reduced to 5:1 despite a higher yield. For comparison, the reported selectivity in the original paper was 25:1.[2] As a result, the undivided system may have a modest but deleterious impact on selectivity.

Direct trapping of the by-products of the TES oxidation by the substrate is likely not a significant factor as this accounts for less than 1% of the product distribution., which must be due to the low amount of [TES^+] species compared to TMSCl, and the bulkiness of it.

In any case, the fact that Gen2 conditions offer higher selectivity and a yield matching the original divided cell conditions[2] seems to indicate that the selectivity drop is not intrinsic to the undivided system.

Optimisation of the "Generation 2 conditions"

Procedure for the optimisation of "Generation 2 conditions"

Under air, a 40 mL dried Schlenk tube equipped with a PTFE stir bar was charged with Bu_4NBr (241 mg, 0.75 mmol, 1.5 eq.) and other electrolytes (if indicated). MeCN (non-anhydrous grade, 2.5 ml or 5.0 mL) was added, followed by the silanes, additives and 4-fluorotrifluoromethylbenzene **1a** (0.500 mmol, 63.0 µL, 1.00 eq.)(if liquid) via micropipette. The tube was fitted with a polypropylene stopper with two crocodile clips holding a Stainless Steel grating (3x2 cm) cathode and a graphite plate anode.

Electrolysis was performed for 25 or 50 mA for the indicating charge with a stir rate of 200 rpm. After electrolysis, the electrodes were rinsed using MeCN, TIPSCl (109 µL, 0.500 mmol, 1.00 eq.) was added as a fluoride scavenger, and C_6F_6 (20 µL) was added for ^{19}F NMR yield assay.

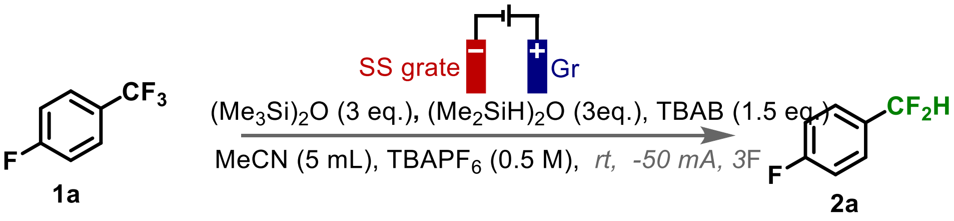

Table S3: Optimisation of the "Generation 2" hydrodefluorination, with $TBAPF_6$ as the electrolyte in 5 mL MeCN. [a]Determined by ^{19}F NMR using C_6F_6 as an internal standard. [b] Calculated by measuring the quantity of TIPSF detected (quant. ^{19}F NMR) after the addition of TIPSCl (1 eq) to the reaction after electrolysis. [c]Calculated according to the following formula: $(F_{passed})/(2 \times yield_{CF2X} + 4 \times yield_{CFX2} + 6 \times yield_{CX3})$. [d]Formation of adducts as byproducts.

Entry	Variation	Charge [*F*]	Conversion [%][a]	Yield **2a** [%][a]	Fluoride [mM][b]	Faradaic efficiency[c]
1	None	3	96	57	15	0.9
2	No HMDS	3	64	63	30	0.5
3	TES (3 eq.) not TMDS	3	99	43	25	1.0
4	PHMS (3 eq.) not TMDS, 0.2mL THF	3	90	29	58	0.9
5	HDMS (6 eq.) not 3 eq.	3	98	47	15	0.8
6	PMDS (3 eq.) no HMDS TES (3 eq.) not TMDS 0.2 mL THF	3	93	59	50	0.8
7	20% EtOH	6	0	0		0.0
8	20% IPA	6	<20%	<20%		0.1
9	20% Acetone	6	97[d]	33		0.5
10	None	3.6	98	45	3	0.9
11	No $TBAPF_6$	3.6	83	62	2	0.6

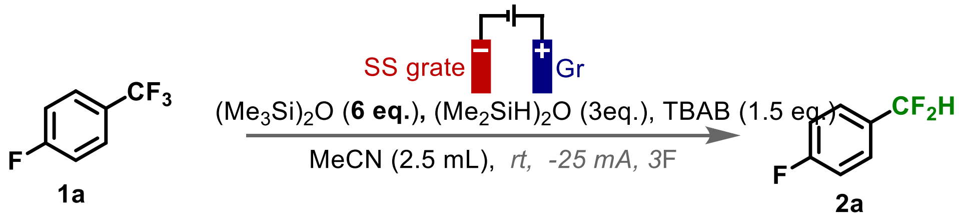

Table S4: Optimisation of the "Generation 2" hydrodefluorination, without $TBAPF_6$, in 2.5 mL MeCN. [a]*Determined by* ^{19}F *NMR using* C_6F_6 *as an internal standard.* [b]*Calculated by measuring the quantity of TIPSF detected (quant.* ^{19}F *NMR) after the addition of TIPSCl (1 eq) to the reaction after electrolysis* [c]*Calculated according to the following formula:* $(F_{passed})/(2 \times yield_{CF2X} + 4 \times yield_{CFX2} + 6 \times yield_{CX3})$. [d]*Formation of adducts as byproducts.*

Entry	Variation	Charge [*F*]	Conversion [%][a]	Yield **2a** [%][a]	Fluoride [mM][b]	Faradaic efficiency[c]
12	MeCN/THF:5/1 as solvent	3	78	63	6	0.6
13	KBr (1.5 eq.) not TBAB KPF_6 (0.1 M)	3	<5%	<5%	66	0.0
14	KBr (1.5 eq.) not TBAB TBAPF6 (0.1 M)	3	78	43	66	0.6
15	LiBr (1.5 eq.) not TBAB TBAPF6 (0.1 eq.)	3	0	0	<1	0.0
16	^{i}PrOTMS (0.5 eq.) in MeCN/THF:5/1	3	73	47	14	0.6
17	MeOTMS (0.5 eq.) in MeCN/THF:5/1	3	62	60	6	0.5
18	^{i}PrOH (0.5 eq.) in MeCN/THF:5/1	3	58	62	2	0.4
19	10% DMC in MeCN/THF:5/1	3	<10%	<10%	<1	0.1
20	10% EC in MeCN[e]	3	86	71	<1	0.7
21	10% PC in MeCN/THF:5/1	3	59	56	2	0.4
22	10% PC in MeCN, no HMDS	3	86	71	6	0.7
23	50% PC in MeCN	3	75	44	<1	0.6
24	PC as solvent	3	86[d]	2	4	Complex mixture

Mechanistic experiments
Impact of bases on the disilylation

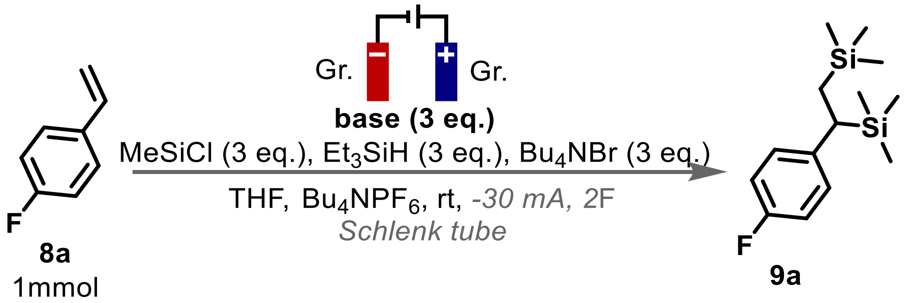

An oven-dried Schlenk tube was cooled under vacuum and charged with Bu_4PF_6 (697 mg, 1.8 mmol, 1.8 eq.), Bu_4NBr (970 mg, 3.0 mmol, 3.0 eq.) and the base (3 eq., dried under vacuum at 80°C overnight) under a positive pressure of N_2, and evacuated/backfilled with N_2 3 times.

4-fluorostyrene (120 µL, 1.00 mmol, 1.00 eq.) was inserted via Gilson pipette by removing the suba seal under a positive pressure of N_2, then anhydrous THF (9 mL), Et_3SiH (0.48 mL, 3.0 mmol, 3.0 eq.) and TMSCl (0.38 mL, 3.0 mmol, 3.0 eq.) were inserted via needle.

Under a positive pressure of N_2, the suba-seal was swapped for polypropylene stopper cap with two corocodile clips holding two graphite plate electropdes.

Electrolysis was performed for 30 mA for 6400 s (2*F*) with a stirring rate of 500 rpm. After electrolysis C_6F_6 (20.0 µL) was added for ^{18}F NMR yield assay.

Table S5: Impact of bases on the metal free disilylation. [a]]Determined by ^{19}F NMR using C_6F_6 as an internal standard

Entry	Base	Conversion [%][a]	Yield **9a** [%][a]	Mass balance [%][a]
1	None (control)	78	64	98
2	K_2CO_3	78	47	91
3	Cs_2CO_3	75	65	109
4	LiOAc	37	2	71
5	Bu_4NOAc	81	17	96
6	$CsCHO_2$	84	50	95

None of the bases tested significantly improved the reaction profile (Table S5). LiOAc (entry 4) lead to a suppression of the reaction, possibly due competitive Li^+ reduction. BuN_4OAc (entry 5) lead to a complex mixture, which may be attributed to the acetate nucleophilicity.

Impact of the amount of Bu_4NBr of the product distribution and Faradaic efficiency in the hydrodefluorination

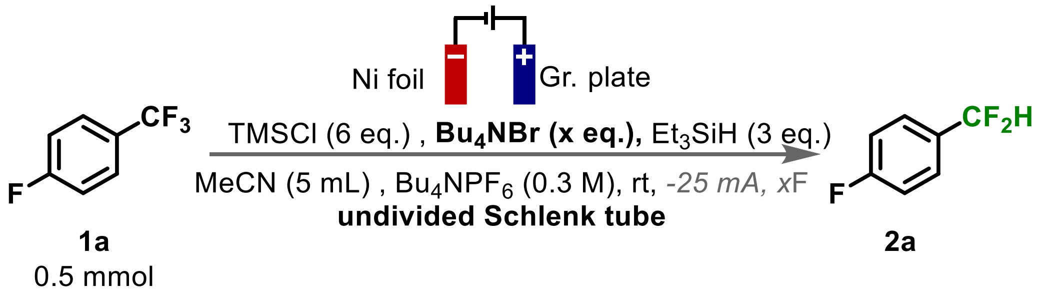

Under a N_2 flow, a 10 mL wide neck oven-dried Schlenk tube equipped with a PTFE stir bar was charged with Bu_4NPF_6 (580 mg, 1.5 mmol, 3.0 eq.) and Bu_4NBr . Anhydrous, degassed MeCN (5 mL) was inserted via syringe, and Et_3SiH (0.24 mL, 1.5 mmol, 3 eq.) and the trifluoromethylarene substrate (0.500 mmol, 1.00 eq., 63 µL) were added via micropipette over an N_2 flow. TMSCl (0.38 mL, 3.0 mmol, 6.0 eq.) was then added via needle. The septum was swapped for a polypropylene stopper with two crocodile clips holding a Ni foil (3x2 cm) cathode and a graphite plate anode.

Electrolysis was performed for 20 mA for 7200 s (3*F*) (unless otherwise specified) with a stir rate of 200 rpm. After electrolysis, the electrodes were rinsed using MeCN and C_6F_6 (20 µL) was added for NMR yield assay.

Table S6: Impact of the amount of Bu_4NBr on the product distribution and the Faradaic efficiency. All yield quoted as ^{19}FNMR vs C_6F_6.[a] FE = $(F_{passed})/(2 \times yield_{CF2X} + 4 \times yield_{CFX2} + 6 \times yield_{CX3})$.

Entry	Bu_4NBr [eq.]	Conversion [%]	$ArCF_2H$ (**2a**) [%]	$ArCF_2TMS$ [%]	$ArCFH_2$ [%]	ArCFHSi [%]	FE[a] [%]
1	0	44	37	3	2	1	*30*
2	0.1	44	40	3	2	1	*32*
3	0.25	45	39	3	1	1	*31*
4	0.75	62	49	5	2	2	*41*
5	1.5	72	58	5	4	2	*50*
6	3	72	57	3	4	2	*48*

Increasing the amount of Bu_4NBr increases the Faradaic efficiency of the reaction, with a maximum at 1.5 eq. This supports the hypothesis of 1 eq. of bromide being able to mediate the generation of 2 eq. of electrons at the anode.

Formation of Et_3SiF with Bu_4NF

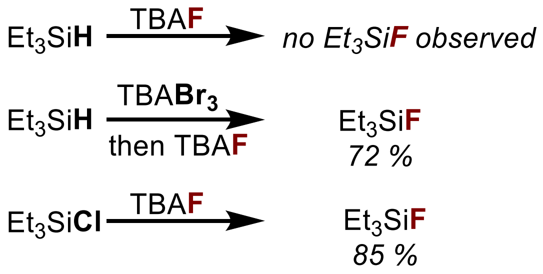

In an NMR tube was inserted MeCN (1 mL), followed by C_6F_6 (1.0 eq.) and either Et_3SiH (0.1 mmol, 1.0 eq.) or Et_3SiCl (1.0 mmol, 1.0 eq.) by micropipette. (If Bu_4NBBr_3 was added, it was added as a solid (48 mg, 1.0 eq.)). The tube was agitated and left standing for 30 min. Subsequently, Bu_4NF (1 M in THF, 0.1 mL, 1.0 eq.) was added via syringe. The tube was agitated and analysed by quantitative ^{19}F NMR after 2 h. Analysis of the spectra (Figure S3) shows clear formation of the TESF adduct at 175.7 ppm (with the characteristic multiplicity) in the reaction of TBAF and Et_3SiCl (bottom spectra,85 %) as well as in the reaction of TBAF and Et_3SiH and $TBABr_3$ (middle spectra, 75 %). In contrast, the reaction without $TBABr_3$ (top spectra) show no Et_3SiF signal.

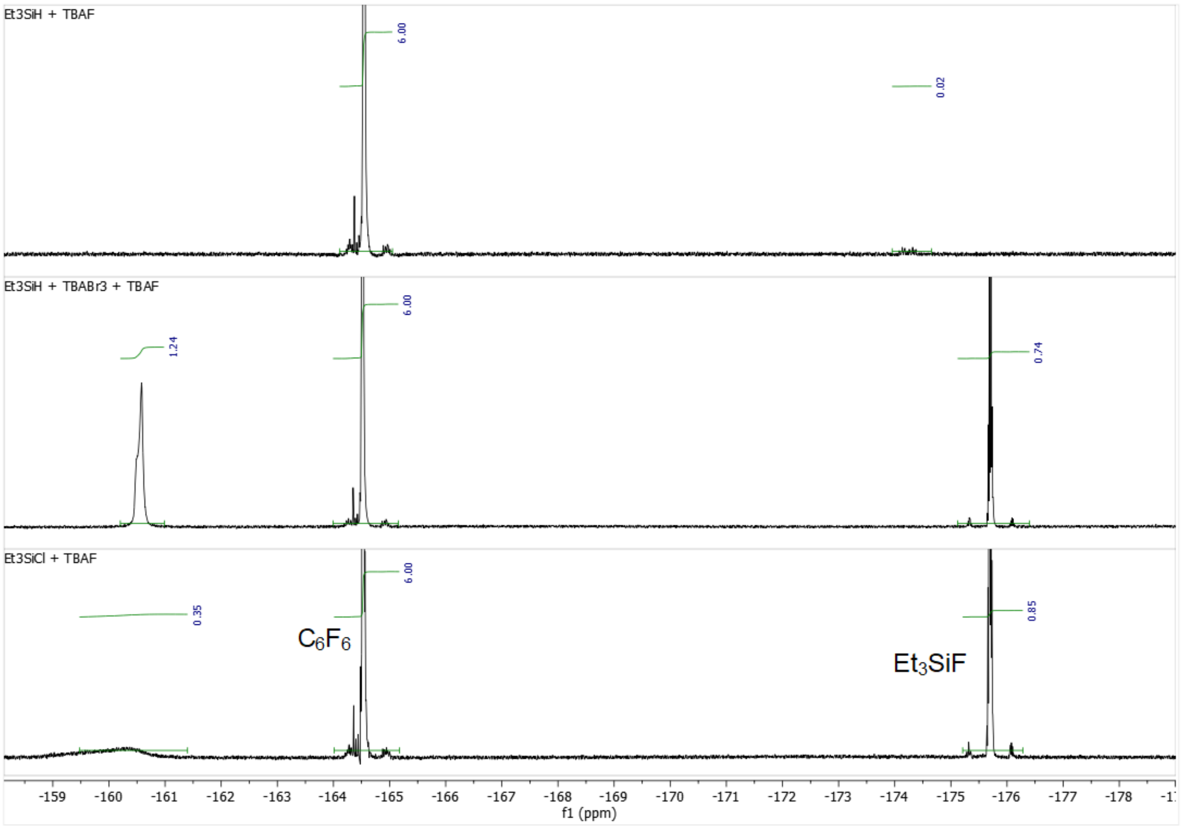

Figure S3: Formation of Et_3SiF in various conditions from Et_3SiH or Et_3SiCl (top to bottom: Et_3SiH+ Bu_4NF; Et_3SiH+Bu_4NBr_3+ Bu_4NF; Et_3SiCl+ Bu_4NF).

Cyclic voltammetry experiments

Each CV was run in 2.5 mL anhydrous MeCN with 0.6 M Bu_4PF_6, using a GC disk electrode at a scan rate of 100 mV/s, unless otherwise specified (see general considerations for full details).

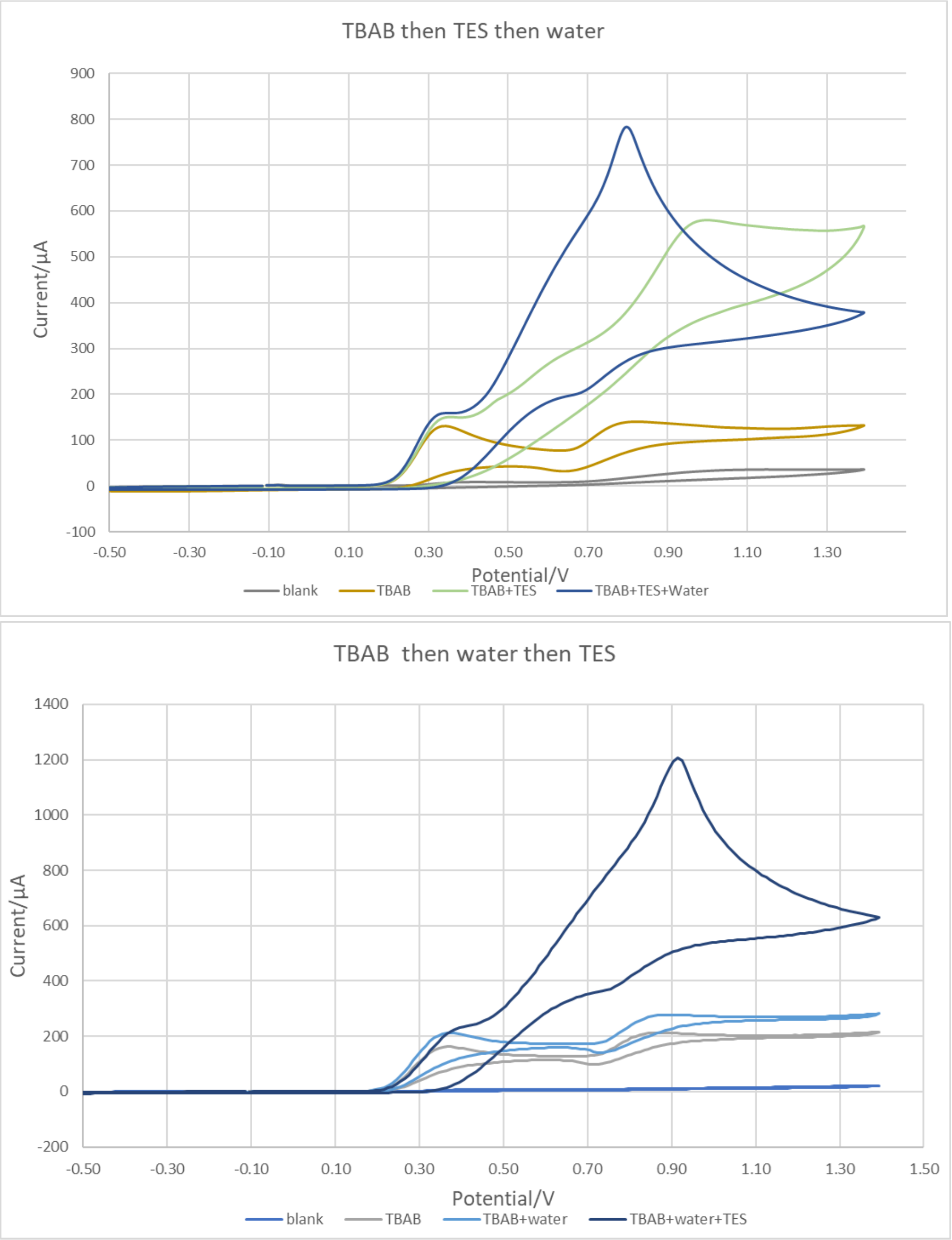

Figure S4: Impact of water and Et_3SiH on the current response of Bu_4NBr oxidation. TES = Et_3SiH; TBAB = Bu_4NBr. 10 mg of each analyte was used.

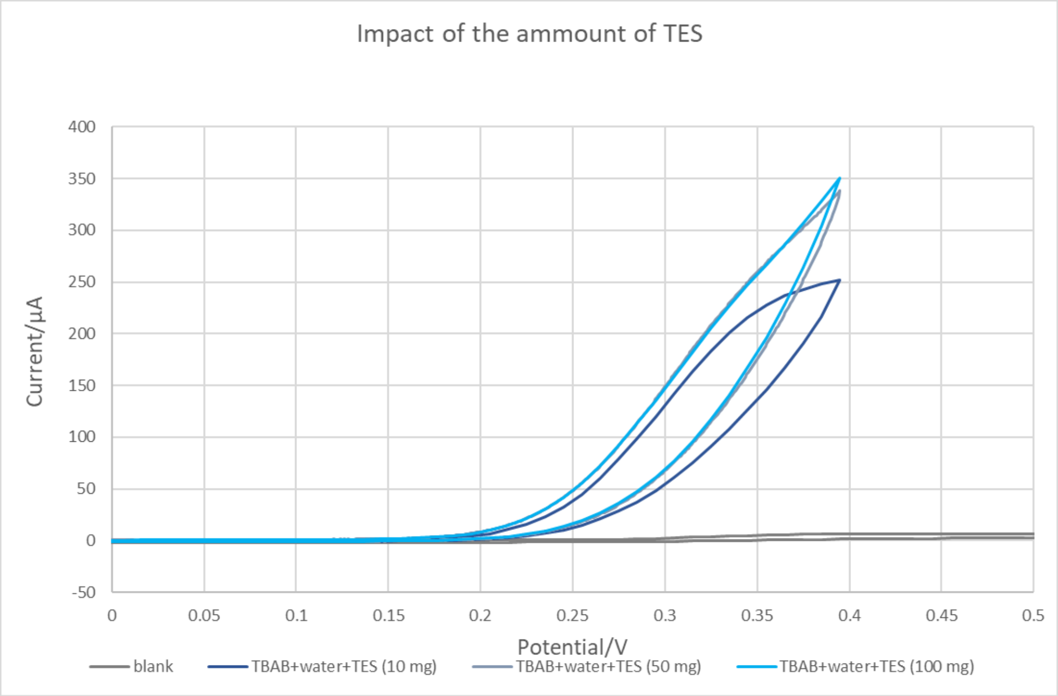

Figure S5: Impact of the amount of Et_3SiH on the current response of Bu_4NBr oxidation. TES = Et_3SiH; TBAB = Bu_4NBr. 10 mg of Bu_4NBr and water used.

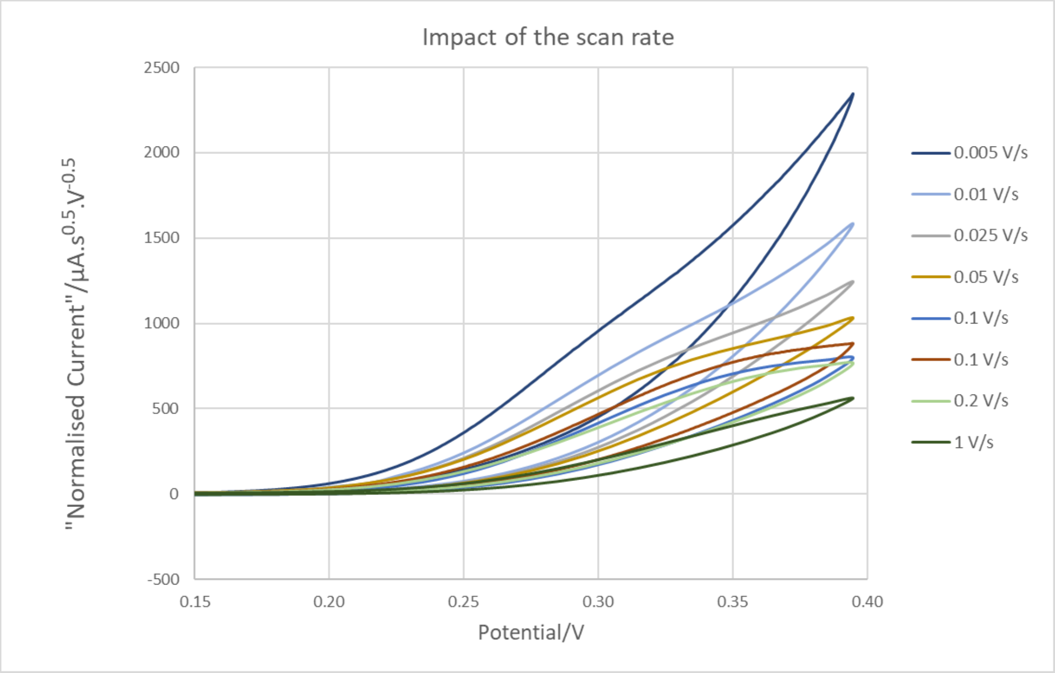

Figure S6: Impact of the scan rate on the normalised current response of Bu_4NBr oxidation. The two 0.1 V/s scans are repeats to ensure there was no significant change or drifting due to concentration change or solvent evaporation; the blue trace was acquired before all the other scan rates, and the orange afterwards.

As shown in Figure S4, the addition of water has a drastic impact on the current response of bromide oxidation above 0.5 V, but only in the presence of silane. This observation supports the hypothesis of a more efficient regeneration of bromide due to hydrolysis of the formed Et_3SiBr in the presence of water. This is consistent with the need for only a catalytic amount of Bu_4NBr in aqueous media (see the pinacol coupling results).

As shown in Figure S5, increasing the amount of silane has a small but noticeable effect on the current response of the first feature of the oxidation of bromide to tribromide. This supports the hypothesis of regeneration of bromide from “oxidised bromide” by the silane, albeit slowly at CV time scales.

Figure S6 shows that once normalised according to the Randles–Sevcik equation (by dividing by the square root of the scan rate), the current responses at different scan rates are not equal. The lower scan rates show a greater normalised current response than the faster ones. This is consistent with the hypothesis of the regeneration of bromide on the CV timescale: at slower scan rates more bromide is regenerated over the course of the experiment leading to higher current responses.

As the current response for the second oxidation peak is much greater in the presence of silane, Figure S4, the regeneration of bromide from bromine is significantly faster.

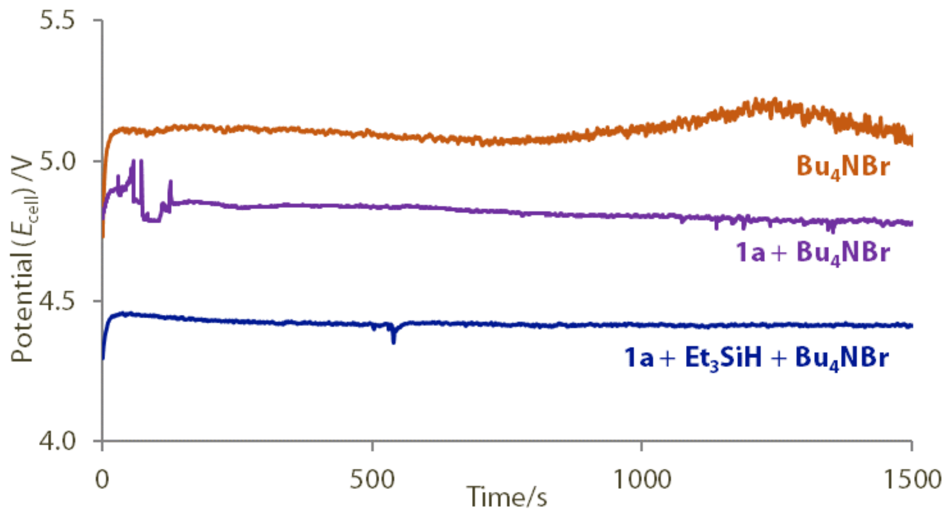

*Figure S7: Bulk electrolysis traces for the trifluoromethylarenes hydrodefluorination. The potentials shown are E_{cell}. Deviations from the General Procedure 1 below for substrate **1a**: “Bu_4NBr”: electrolysis ran without Et_3SiH or substrate **1a**; “**1a** + Bu_4NBr”: electrolysis ran without Et_3SiH; “**1a** + Et_3SiH + Bu_4NBr”: No deviation.*

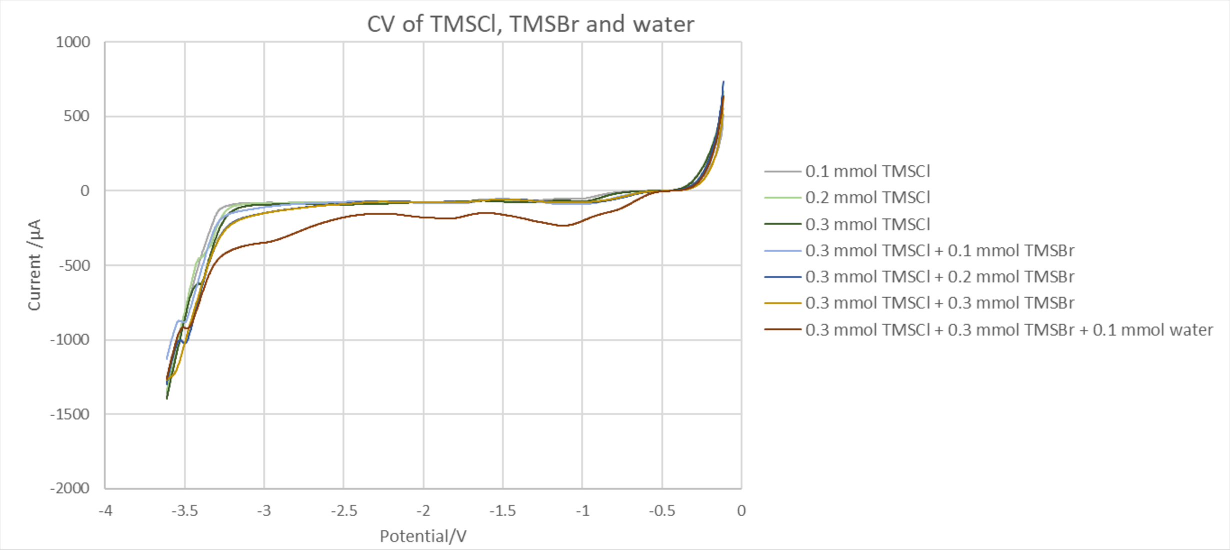

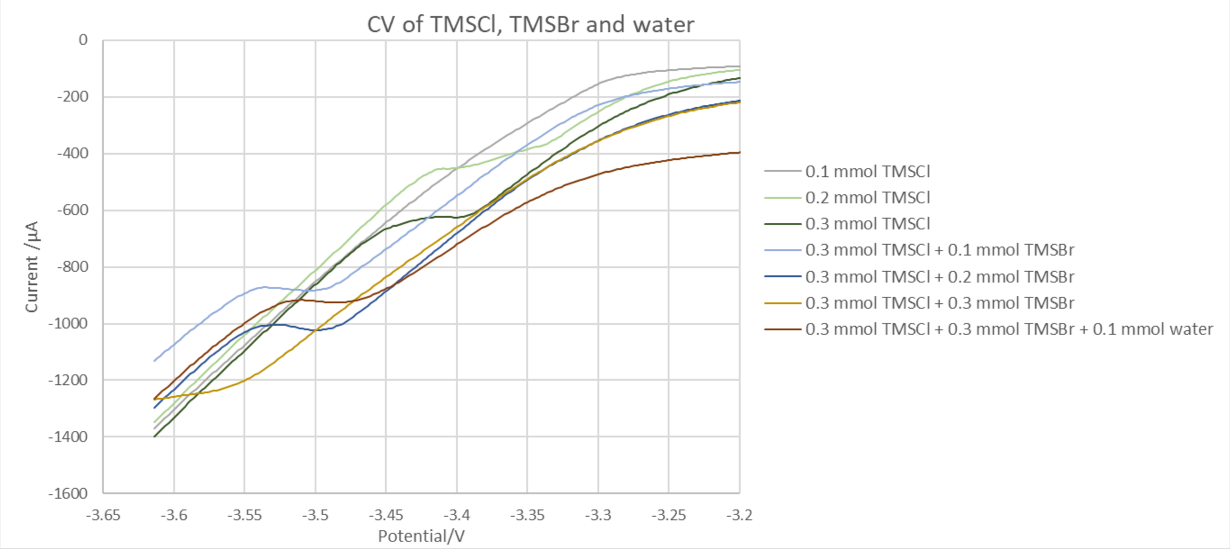

Figure S8: CV experiment of TMSCl, TMSBr, and water. Each CV was run in 3mL anhydrous MeCN with 0.4 M Bu_4PF_6, using a Ni disk electrode at a scan rate of 100 mV/s. Only forward scan shown.

CV experiment showed that TMSCl only displays a reduction feature at very negative potentials (peak at −3.3 V - −3.4 V vs Fc/Fc$^+$, Figure S8), very close to the edge of the solvent windows.

Addition of TMSBr increased the current response in that range, indicating a very close reduction potential of these two species. Addition of water reduced the intensity of this peak, indicating that this feature is intrinsic to TMSBr and TMSCl, and is not due to hydrolysis product reduction.

As a result, generation of TESBr should not affect the reduction process.

Oxidative stability to Br_3^- with or without TES:

To assess the tolerance of functional groups to the new process and in particular oxidatively sensitive groups, we conducted a set of experiments in which different compounds were subjected to Bu_4NBr_3 in the presence and absence of Et_3SiH (TES).

General procedure:

Two solutions of each substrate (see list in the table below) were prepared: the substrate 0.50 mmol (1 eq., 1.0 M) and C_6F_6 (9.6 µL, 0.083 mmol, 0.17 eq., internal standard) were dissolved in a mixture of MeCN (0.50 mL) and THF (0.20 mL). For each substrate, Et_3SiH (160 µL, 1.0 mmol, 1.0 eq.) was added to one of the solutions. For solubility reasons, 0.50 mL of THF was added to each solution of PPh_3, and 0.20 mL of water was added to each solution of hydroquinone.

A solution of Bu_4NBr_3 (9.6 g, 20 mmol) and CH_2Br_2 (352 µL, 5.00 mmol, as ^1H NMR standard) in MeCN (15.0 mL) was prepared. After sonication, the total volume was 22.3 mL. 0.75 mL of this solution was added over 1 min to each of the substrate solutions via syringe (corresponding to 1.3 eq. Bu_4NBr_3 and 0.34 eq. CH_2Br_2). The resulting solutions were shaken and sonicated for 5 minutes, then left to stand for 18 h. 100 µL of each solution was transferred to an NMR tube (if solid were present, only the supernatant was pipetted) and diluted with 0.5 mL of $CDCl_3$ before being analysed by ^1H NMR and ^{19}F NMR spectrometry.

Table S7: Stability of various electron-rich or easily oxidisable substrates to Br_3^- in the presence or absence of Et_3SiH. Recovery was determined by ^{19}F NMR or ^{1}H NMR.

Entry	Substrate	Recovery of substrate with Bu_4NBr_3 [%]	Recovery of substrate with Bu_4NBr_3 and Et_3SiH [%]	Notes
1	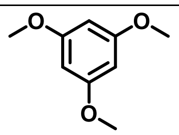 *trimethoxybenzene*	<20 %	83 %	
2	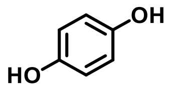 *hydroquinone*	0 %	80%	
3	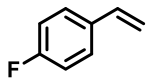 *4-fluorostyrene*	0 % (92 % mass balance for aromatic signals)	87%	
4	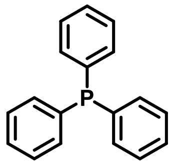 *triphenylphosphine*	42%	70%	Formation of precipitate
5	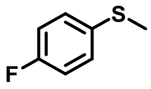 *4-fluorothioanisole*	Quant.	Quant.	
6	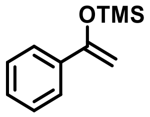 *1-phenyl-1-trimethylsiloxyethylene*	0% (39 % mono- and 38 % di-brominated)	0% (41% acetophenone, 25% mono-brominated)	
7	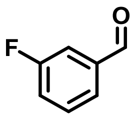 *3-fluorobenzaldehyde*	Quant.	33 %	

For entries 1-4 (electron-rich rings, alkenes and phosphine) TES protected the substrate against bromination. For entry 5, the thiophenyl ether was not affected under either conditions. For entry 6, TES partly protected the silyl enol ether against bromination. For entry 7, reduction of the aldehyde by TES was observed.

Faradaic efficiency of the reaction:

*Table S8: Comparison of the Faradaic Efficiency (FE) between the undivided silane/br system and the original report. Calculated by FE = Yield * $F_{theoretical\ requiered}/F_{passed}$). HDF = Hydrodefluorination.HDF and Ketone HDF: theoretical 2F required, original report passed 2F. Disylation: theoretical 2F required, original report passed 3F. Acetylation: theoretical 2F required, original report passed 3.1F. Disylation: theoretical 2F required, original report passed 3.5F (5F for **9C**). Pinacol: theoretical 1F requiered, original report with thiourea used 2F, report with Sm(+) used 2.1F, report with Sn(+) did not report charge.*

Reaction	Substrate	FE silane/bromide [%]	FE original report [%]
HDF	**2a**	43	76
HDF	**2b**	37	80
HDF	**2c**	32	58
HDF	**2d**	37	63
HDF	**2e**	35	64
HDF	**2f**	20	64
HDF	**3a**	32	45
Ketone-HDF	**5a**	28	97
Acylation	**7a**	33	31
Acylation	**7b**	26	21
Acylation	**7c**	62	41
Acylation	**7d**	46	40
Disylation	**9a**	56	50
Disylation	**9b**	61	56
Disylation	**9c**	34	20
Disylation	**9d**	64	39
Pinacol	**10a**	54	41 (thiourea), 38 (Sm)
Pinacol	**10b**	56	35 (thiourea)
Pinacol	**10c**	48	31 (thiourea), 30 (Sm),
Pinacol	**10d**	45	44 (thiourea), 45 (Sm)
Pinacol	**10e**	49	38 (thiourea)

As shown in Table S8, Faradaic efficiency of the hydrodefluorination is lower in the silane/Br system. However, for the acylation, disylation and pinacol, the FE is better for the silane/Br system

Undivided trifluoromethylarenes hydrodefluorination (General procedure 1)

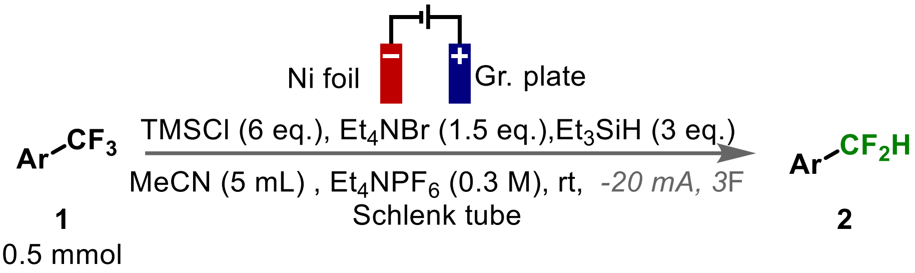

Under a N_2 flow, a 10 mL wide neck oven-dried Schlenk tube equipped with a PTFE stir bar was charged with Et_4NPF_6 (421 mg, 1.5 mmol, 3.0 eq.), Bu_4NBr (241 mg, 0.75 mmol, 1.5 eq.) and the trifluoromethylarene substrate (0.500 mmol, 1.00 eq.)(if solid). Anhydrous, degassed MeCN (5 mL) was inserted via syringe, and Et_3SiH (0.24 mL, 1.5 mmol, 3 eq.) and the trifluoromethylarene substrate (0.500 mmol, 1.00 eq.)(if liquid) were added via micropipette over an N_2 flow. TMSCl (0.38 mL, 3.0 mmol, 6.0 eq.) was then added via needle. The septum was swapped for a polypropylene stopper with two crocodile clips holding a Ni foil (3x2 cm) cathode and a graphite plate anode.

Electrolysis was performed for 20 mA for 7200 s (3*F*) (unless otherwise specified) with a stir rate of 50 rpm. After electrolysis, the electrodes were rinsed using MeCN and C_6F_6 (20 µL) was added for NMR yield assay. Compound **2c** was further purified while **2a**, **2b, 2e, 2f, 3a** were not.

Cathode	Anode	Stir rate	Current	Duration	Charge
Ni foil	graphite plate	50 rpm	20 mA	7200 s	3*F*

Compounds synthesised using General Procedure 1

4-Fluoro-(difluoromethyl)benzene, 2a

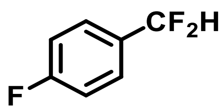

2a was synthesised from 4-fluorobenzotrifluoride using General Procedure 1. Because of its volatility, it was not isolated: the ^{19}F NMR yield was determined to be 64%. The following diagnostic NMR signals were observed in the crude reaction mixture:

^{1}H NMR (400 MHz, Acetonitrile) δ 7.64 – 7.56 (m, 2H), 7.23 (t, J = 8.9 Hz, 2H), 6.80 (t, J = 56.1 Hz, 1H).
^{19}F NMR (376 MHz, Acetonitrile) δ -110.0 (d, *J* = 56.0 Hz, 2F), -111.0 – -111.2 (m, 1F).
Data in agreement with the literature.[2]

4-Fluoro-(fluoromethyl)benzene, 3a

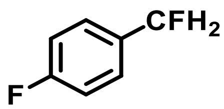

3a was synthesised from 4-Fluoro-(difluoromethyl)benzene (**2a**) using General Procedure 1. Bu_4NF (1 M in THF, 5.0 mL, 5.0 mmol, 10 eq.) was added to the crude reaction mixture after electrolysis. Because of its volatility, it was not isolated: the ^{19}F NMR yield was determined to be 48%. The following diagnostic NMR signals were observed in the crude reaction mixture:

^{1}H NMR (400 MHz, Acetonitrile) δ 5.33 (d, *J* = 48.0 Hz).
^{19}F NMR (376 MHz, Acetonitrile) δ -114.4 – -114.6 (m, 1F), -203.8 (t, *J* = 49.3, 48.4 Hz, 1F).
Data in agreement with the literature.[2]

(Difluoromethyl)benzene, 2b

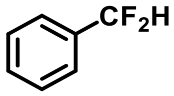

2b was synthesised from benzotrifluoride using General Procedure 1 with 4*F*. Because of its volatility, it was not isolated: the ^{19}F NMR yield was determined to be 73%. The following diagnostic NMR signals were observed in the crude reaction mixture:

^{1}H NMR (400 MHz, Acetonitrile) δ 7.58 – 7.48 (m, 6H), 6.79 (t, *J* = 56.2 Hz, 1H).
^{19}F NMR (377 MHz, Acetonitrile) δ -110.2 (d, J = 56.2 Hz).
Data in agreement with the literature.[2]

Ethyl 4-(difluoromethyl)benzoate, 2c

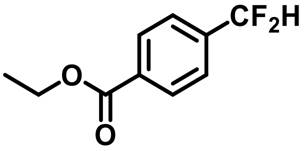

2c was synthesised from 4-(trifluoromethyl)benzoate using General Procedure 1. After concentration under reduced pressure, Bu_4NF (1M in THF, 1 mL, 1 mmol, 2 eq.) was added to the crude reaction mixture. The mixture was agitated for a few minutes, loaded unto silica by concentrating it under reduced pressure and purified by FCC to yield a colourless oil (53 mg, 53% (9:1 **2c**: over-reduced **3c**), which represents a yield of 47% of **2c** when correcting for 10% contamination with **3c**, as also observed in the divided cell conditions).

^{1}H NMR (301 MHz, Chloroform-*d*) δ 8.13 (dd, J = 7.9, 0.9 Hz, 2H), 7.58 (d, J = 8.2 Hz, 2H), 6.69 (t, J = 56.1 Hz, 1H), 4.40 (q, J = 7.1 Hz, 2H), 1.40 (t, J = 7.1 Hz, 3H).
^{19}F NMR (283 MHz, Chloroform-*d*) δ -112.0 (d, J = 55.8 Hz).
^{13}C NMR (101 MHz, Chloroform-*d*) δ 165.8, 138.4 (t, *J* = 22.4 Hz), 132.7, 129.9, 125.6 (t, *J* = 6.0 Hz), 114.0 (t, *J* = 239.7 Hz), 61.3, 14.3.
Data in agreement with the literature.[2]

1-(Benzyloxy)-3-(difluoromethyl)benzene, 2d

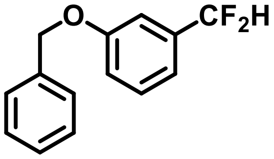

2d was synthesised from 1-(benzyloxy)-3-(trifluoromethyl)benzene using General Procedure 1. After concentration under reduced pressure and loading unto silica, it was purified by FCC to yield a colourless oil (64 mg, 55 %)

^{1}H NMR (400 MHz, Chloroform-*d*) δ 7.52 – 7.32 (m, 7H), 7.16 – 7.03 (m, 3H), 6.60 (t, *J* = 56.5 Hz, 1H), 5.09 (s, 2H).
^{19}F NMR (377 MHz, Chloroform-*d*) δ -110.6 (d, *J* = 56.5 Hz).
^{13}C NMR (126 MHz, Chloroform-d) δ 159.2, 136.7, 136.0 (t, J = 22.3 Hz), 130.1, 128.8, 128.3, 127.7, 118.3 (t, J = 6.2 Hz), 117.5 (t, J = 2.0 Hz), 114.7 (t, J = 239.1 Hz), 111.9 (t, J = 6.2 Hz), 70.3
Data in agreement with the literature.[2]

1-(Difluoromethyl)-4-methoxybenzene, 2e

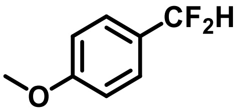

2e was synthesised from 1-(trifluoromethyl)-4-methoxybenzene using General Procedure 1. Because of its volatility, it was not isolated: the ^{19}F NMR yield was determined to be 53%. The following diagnostic NMR signals were observed in the crude reaction mixture:

^{1}H NMR (400 MHz, Acetonitrile) δ 6.46 (t, J = 56.6 Hz).
^{19}F NMR (377 MHz, Acetonitrile) δ -108.3 (d, J = 56.8 Hz).
Data in agreement with the literature. [2]

1-(Difluoromethyl)-4-(dimethylamino)benzene, 2f

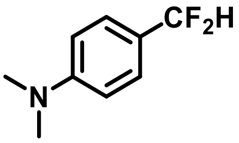

2f was synthesised from 1-(trifluoromethyl)-4-(dimethylamino)benzene using General Procedure 1 with 6*F*. Because of its volatility, it was not isolated: the ^{19}F NMR yield was determined to be 60%. The following diagnostic NMR signals were observed in the crude reaction mixture:

^{1}H NMR (400 MHz, Acetonitrile/Chloroform-*d*) δ 7.88 (d, *J* = 8.4 Hz, 2H), 7.59 (d, *J* = 8.4 Hz, 2H), 6.62 (t, *J* = 55.9 Hz, 1H).
^{19}F NMR (376 MHz, Acetonitrile/Chloroform-*d*) δ -112.0 (d, J = 56.0 Hz).
Data in agreement with the literature. [2]

Undivided trifluoromethylketone hydrodefluorination

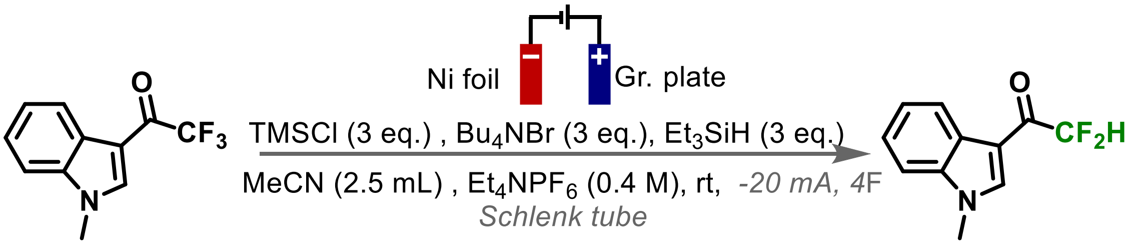

4a 0.5 mmol — **5a** 55%

Under a N_2 flow, a 10 mL wide neck oven-dried Schlenk tube equipped with a PTFE stir bar was charged with Et_4NPF6 (275 mg, 1 mmol, 2.0 eq.), 1-methyl-3-trifluoroacetylindole (0.500 mmol, 1.00 eq.) and Bu_4NB (483 mg, 1.5 mmol, 3 eq.). Anhydrous, degassed MeCN (2.5 mL) was inserted via syringe and Et_3SiH (0.24 mL, 1.5 mmol, 3 eq.) was added via micropipette under a N_2 flow. TMSCl (0.38 mL, 3.0 mmol, 6.0 eq.) was added via needle. The septum was swapped for a polypropylene stopper with two crocodile clips holding a Ni foil (3 x 2 cm) cathode and a graphite plate anode.

Electrolysis was performed for 20 mA for 9600 s (4*F*) with a stir rate of 50 rpm. After electrolysis, the electrodes were rinsed using MeCN and C6F6 (20 µL) was added for NMR yield assay (^{19}F NMR = 64%). Evaporation of the volatile under reduced pressure was followed by FCC (5-15% EtOAc in pentane) to afford a white solid (55%).

^{1}H NMR (400 MHz, Chloroform-*d*) δ 7.4 – 7.3 (m, 3H), 6.1 (t, *J* = 54.3 Hz, 1H), 3.9 (s, 3H), 8.4 – 8.4 (m, 1H), 8.0 (t, *J* = 1.8 Hz, 1H).
^{19}F NMR (377 MHz, Chloroform-*d*) δ -120.1 (dd, *J* = 54.2, 1.8 Hz).
^{13}C NMR (101 MHz, Chloroform-*d*) δ 182.8 (t, J = 25.1 Hz), 137.9 (t, J = 7.1 Hz), 137.2, 126.9, 124.3, 123.6, 122.5 112.2 (t, J = 254.2 Hz), 110.4 (t, J = 2.3 Hz), 109.9, 33.8.
Data in agreement with the literature.[1]

Cathode	Anode	Stir rate	Current	Duration	Charge
Ni foil	graphite plate	200 rpm	20 mA	9600 s	4*F*

Undivided acetophenones acetylation (General procedure 2)

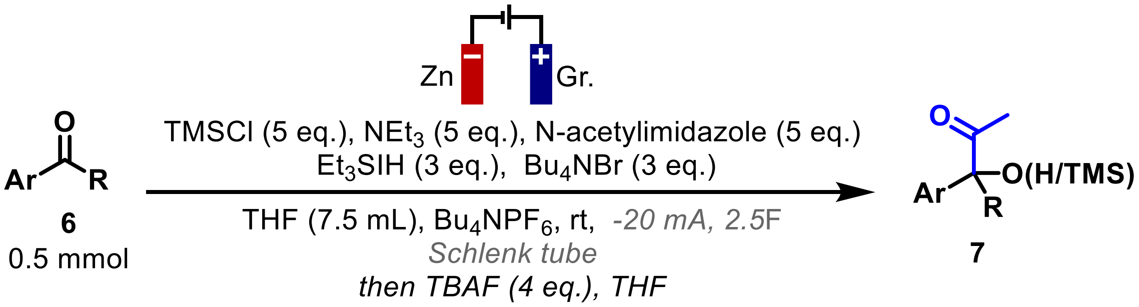

Under a N_2 flow, a 40 mL oven-dried Schlenk tube equipped with a PTFE stir bar was charged with N-acetylimidazole (275 mg, 2.50 mmol, 5.0 eq.), Bu_4NPF_6 (870mg, 2.25 mmol, 0.3 M), Bu_4NBr (403 mg, 1.25 mmol, 2.5 eq.) and the substrate (0.500 mmol, 1.00 eq.)(if solid). Anhydrous THF (7.5 mL) was inserted via needle, and the substrate (0.500 mmol, 1.00 eq.)(if liquid), Et_3N (0.34 mL, 2.50 mmol, 5.0 eq.) and Et_3SiH (0.20 mL, 1.25 mmol, 2.5 eq.) were added via pipetting under a strong N_2 pressure. TMSCl (0.30 mL, 2.50 mmol, 5.0 eq.) was added via needle. The septum was swapped for a polypropylene stopper with two crocodile clips holding a Zn plate cathode and a graphite plate anode.

Electrolysis was performed for 20 mA for 6000 s (2.5*F*) with a stir rate of 200 rpm. After electrolysis, the electrodes were rinsed using MeCN. The reaction mixture was then concentrated under reduced pressure. The crude was handled differently depending on the desired product:

For **7c,** the solids were triturated and filtered off with diethyl ether (2 x 25 mL) then EtOAc (25 mL). The residue was concentrated and dissolved in a minimum amount of EtOAc and purified by FCC (pure pentane, liquid injection in EtOAc).

For **7a, 7b,** and **7d**, the solids were dissolved in THF (50 mL) and cooled to 0°C. Bu_4NF (2.0 mL, 1 M in THF, 2.0 mmol, 4.0 eq.) was added and the mixture was stirred for 1.5 h. Loading onto silica by concentration under reduced pressure and FCC afforded the desired compounds.

Cathode	Anode	Stir rate	Current	Duration	Charge
Zn plate	graphite plate	200 rpm	20 mA	6000s	2.5*F*

3-(4-Fluorophenyl)-3-hydroxybutan-2-one, 7a

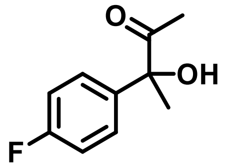

Synthesised according to General Procedure 2. FCC (20% EtOAc in pentane) afforded a faint yellow gel (41%).

^{1}**H NMR** (400 MHz, Chloroform-*d*) δ 7.41 (dd, *J* = 8.9, 5.2 Hz, 2H), 7.05 (dd, *J* = 8.9, 8.5 Hz, 2H), 4.50 (br s, 1H), 2.07 (s, 3H), 1.76 (s, 3H).
^{19}**F NMR** (377 MHz, Chloroform-*d*) δ -114.3 (tt, *J* = 8.3, 5.2 Hz).
^{13}**C NMR** (101 MHz, Chloroform-*d*) δ 209.5, 162.6 (d, *J* = 247.4 Hz), 137.4 (d, *J* = 3.1 Hz), 128.0 (d, *J* = 8.3 Hz), 115.7 (d, *J* = 21.5 Hz), 79.6, 24.4, 23.5.
Data in agreement with the literature.[3]

3-Hydroxy-3-(4-methoxyphenyl)butan-2-one, 7b

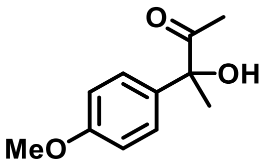

Synthesised according to General Procedure 2. FCC (20-60% EtOAc in pentane) was followed by reverse phase preparative HPLC and concentration of the fractions under reduced pressure with coevaporation with MeCN to afford a yellow oil (33%).

^{1}**H NMR** (400 MHz, Chloroform-*d*) δ 7.38 – 7.30 (m, 2H), 6.93 – 6.86 (m, 2H), 4.51 (s, 1H), 3.81 (s, 3H), 2.07 (s, 3H), 1.76 (s, 2H).
^{13}**C NMR** (101 MHz, Chloroform-*d*) δ 209.9, 159.5, 133.6, 127.4, 114.1, 79.5, 55.4, 24.1, 23.4.
Data in agreement with the literature.[3]

3-Phenyl-3-(trimethylsilyloxy)butan-2-one, 7c

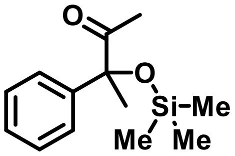

Synthesised according to General Procedure 2. FCC (pure pentane) afforded a clear oil (77%).

^{1}**H NMR** (400 MHz, Chloroform-*d*) δ 7.43 – 7.38 (m, 2H), 7.37 – 7.29 (m, 2H), 7.29 – 7.21 (m, 1H), 2.04 (s, 3H), 1.69 (s, 3H), 0.17 (s, 7H).
^{13}**C NMR** (101 MHz, Chloroform-*d*) δ 210.1, 143.4, 128.4, 127.5, 125.1, 83.6, 25.8, 24.1, 2.1.
Data in agreement with the literature.[3]

3-Hydroxy-3-phenylhexan-2-one, 7d

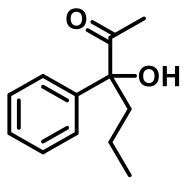

Synthesised according to General Procedure 2. FCC (20% EtOAc in pentane) afforded a faint yellow gel (57%).

^{1}H NMR (500 MHz, Chloroform-*d*) δ 7.52 – 7.47 (m, 2H), 7.42 – 7.36 (m, 2H), 7.34 – 7.30 (m, 1H), 4.56 (s, 1H), 2.23 – 2.15 (m, 2H), 2.11 (s, 3H), 1.59 – 1.48 (m, 1H), 1.26 – 1.14 (m, 1H), 1.01 (t, *J* = 7.4 Hz, 3H).
^{13}C NMR (126 MHz, Chloroform-*d*) δ 209.5, 141.0, 128.6, 127.9, 126.2, 82.6, 38.7, 23.6, 16.6, 14.4.
Data in agreement with the literature.[4]

Metal-free disylation of styrenes

Metal-free disylation of styrenes in a Schlenk tube (General procedure 3)

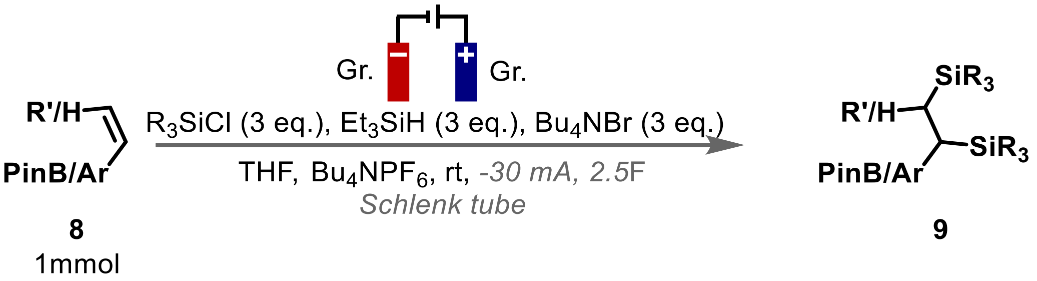

A 40 mL oven-dried Schlenk tube was taken into a nitrogen-filled glovebox and charged with Bu_4NPF_6 (700 mg, 1.8 mmol, 1.8 eq.) and Bu_4NBr (970 mg, 3.0 mmol, 3.0 eq.). Anhydrous THF (9 mL), the substrate (1.00 mmol, 1.00 eq.), Et_3SiH (0.48 mL, 3.0 mmol, 3.0 eq.) and the trialkylsilane (3.0 mmol, 3.0 eq.) were added. The tube was closed with a polypropylene stopper with two crocodile clips holding graphite plates.
Electrolysis was performed for 30 mA for 8000 s (2.5*F*). After electrolysis, the stopper was swapped for a septum and the reaction tube was taken out of the glovebox and connected to a N_2 Schlenk line. Ethanolamine (0.30 mL, 5.0 mmol, 5.0 eq.) was added via syringe and the mixture was stirred under N_2 for an additional hour.

The reaction mixture was then filtered through a 25 mL silica plug and eluted with additional solvent, concentrated under reduced pressure, loaded unto silica gel, and purified by FCC to afford the title compounds.

Cathode	Anode	Stir rate	Current	Duration	Charge
graphite plate	graphite plate	500 rpm	30 mA	8000s	2.5*F*

(1-(4-Fluorophenyl)ethane-1,2-diyl)bis(trimethylsilane), 9a

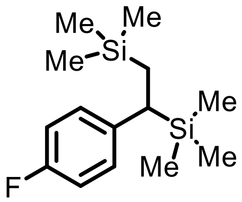

Following General Procedure 3, after a plug with 5% Et_2O/pentane (2 x 100 mL), concentration under reduced pressure and FCC (pure pentane), a clear oil was obtained (70%).

^{1}H NMR (400 MHz, Chloroform-*d*) δ 6.96 (ddd, *J* = 8.4, 5.4, 2.6 Hz, 1H), 6.90 (t, *J* = 8.8 Hz, 1H), 2.06 (dd, *J* = 13.0, 2.5 Hz, 1H), 1.00 (dd, *J* = 15.1, 13.0 Hz, 1H), 0.88 – 0.77 (m, 1H), -0.08 (d, *J* = 0.7 Hz, 5H), -0.20 (d, *J* = 0.7 Hz, 5H).
^{19}F NMR (377 MHz, Chloroform-*d*) δ -119.8 (tt, *J* = 8.7, 5.4 Hz).
^{13}C NMR (101 MHz, Chloroform-*d*) δ 160.5 (d, *J* = 241.4 Hz), 140.9 (d, *J* = 3.1 Hz), 128.9 (d, *J* = 7.5 Hz), 114.8 (d, *J* = 20.8 Hz), 30.9, 16.3, -0.9, -3.3.
^{29}Si NMR (400 MHz, Chloroform-*d*) δ 4.8, 3.4.
Data in agreement with the literature [5]

3-Hydroxy-3-phenylhexan-2-one, 9b

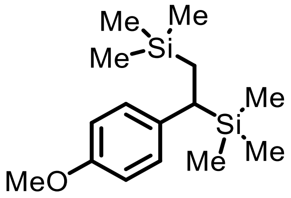

Following General Procedure 3, after a plug with pure Et_2O (2 x 100 mL), concentration under reduced pressure and FCC (pure pentane), a clear oil was obtained (76 %).

^{1}**H NMR** (400 MHz, Chloroform-*d*) δ 6.94 – 6.88 (m, 2H), 6.78 – 6.72 (m, 2H), 3.76 (s, 3H), 2.00 (dd, *J* = 13.1, 2.4 Hz, 1H), 0.99 (dd, *J* = 15.0, 13.1 Hz, 1H), 0.85 – 0.76 (m, 1H), -0.09 (s, 9H), -0.21 (s, 9H).
^{13}**C NMR** (101 MHz, Chloroform-*d*) δ 156.8, 137.1, 128.6, 113.4, 55.2, 30.4, 16.2, -1.0, -3.2.
Data in agreement with the literature [5]

4-(1,2-Bis(trimethylsilyl)ethyl)-N,N-dimethylaniline, 9c

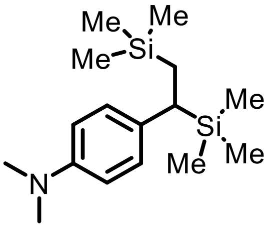

Following General Procedure 3 with 4.0 eq. of chlorotrimethylsilane, after a plug with pure Et_2O (2 x 100 mL), concentration under reduced pressure and FCC (2% EtOAc in pentane), a clear oil was obtained (43 %).

^{1}**H NMR** (400 MHz, Chloroform-*d*) δ 6.91 – 6.85 (m, 2H), 6.67 – 6.59 (m, 2H), 2.88 (s, 6H), 1.96 (dd, *J* = 13.0, 2.5 Hz, 1H), 0.99 (dd, *J* = 15.0, 13.0 Hz, 1H), 0.85 – 0.77 (m, 1H), -0.09 (s, 9H), -0.20 (s, 9H).
^{13}**C NMR** (101 MHz, Chloroform-*d*) δ 148.0, 133.5, 128.4, 113.0, 41.1, 30.0, 16.1, -0.9, -3.1.
Data in agreement with the literature [5]

(1-(4,4,5,5-tetramethyl-1,3,2-dioxaborolan-2-yl)ethane-1,2-diyl)bis(triethylsilane), 9d

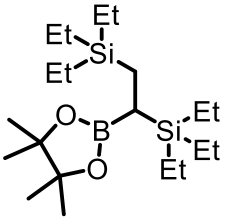

^{1}**H NMR** (400 MHz, Chloroform-*d*) δ 1.22 (s, 6H), 1.21 (s, 6H), 1.03 – 0.85 (m, 21H), 0.76 (dd, *J* = 14.8, 11.9 Hz, 1H), 0.58 (q, *J* = 8.1 Hz, 7H), 0.53 – 0.43 (m, 6H), 0.36 (dd, *J* = 11.9, 1.1 Hz, 1H).
^{11}**B NMR** (128 MHz, Chloroform-*d*) δ 34.4.
^{13}**C NMR** (101 MHz, Chloroform-*d*) δ 82.7, 25.7, 25.0, 7.8, 7.6, 6.9, 5.3, 3.3, 3.2.
Data in agreement with the literature [5]

Metal-free disilylation of 4-fluorostyrene using the ElectraSyn

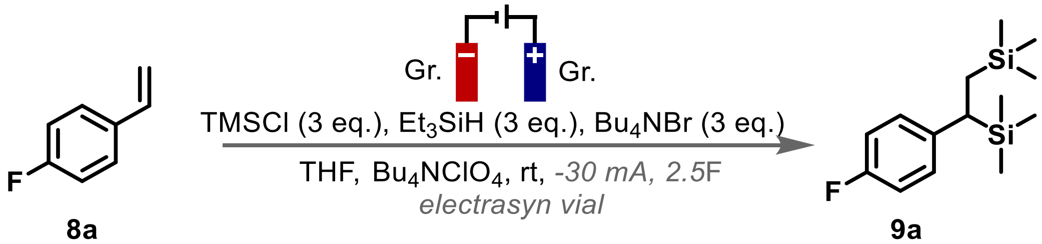

8a 1mmol

9a 72%

An oven-dried 10 mL Electrasyn vial was taken into a nitrogen-filled glovebox and charged with Bu_4NClO_4 (270 mg, 1.1 mmol, 1.1 eq.) and Bu_4NBr (970 mg, 3.0 mmol, 3.0 eq.). The thread of the vial was wrapped in PTFE tape, and the vial was sealed using an ElectraSyn lid fitted with two graphite plates. The vial was taken out and placed under a positive pressure of N_2 via a needle connected to a Schlenk line.

4-fluorostyrene (120 µL, 1.00 mmol, 1.00 eq.) was inserted into a 1-mL Eppendorf and diluted in anhydrous THF (0.5 mL). This solution, followed by anhydrous THF (8.5 mL), Et_3SiH (0.48 mL, 3.0 mmol, 3.0 eq.) and TMSCl (0.38 mL, 3.0 mmol, 3.0 eq.) were inserted via needle via the septum on the lid.

Electrolysis was performed for 30 mA for 6240 s (2.5*F*). After electrolysis, ethanolamine (0.30 mL, 5.0 mmol, 5.0 eq.) was added via syringe and the mixture was stirred under N_2 for an additional hour. Afterwards, the electrodes were rinsed using EtOAc and C_6F_6 (20.0 µL) was added for NMR yield assay (^{19}F NMR = 78%). The reaction mixture was then filtered through a 25 mL silica plug using pentane (50 mL), filtered over cotton, concentrated under reduced pressure, loaded unto silica gel, and purified by FCC (pure pentane) to afford **9a** as clear oil (72%).

Metal-free pinacol coupling (General procedure 4)

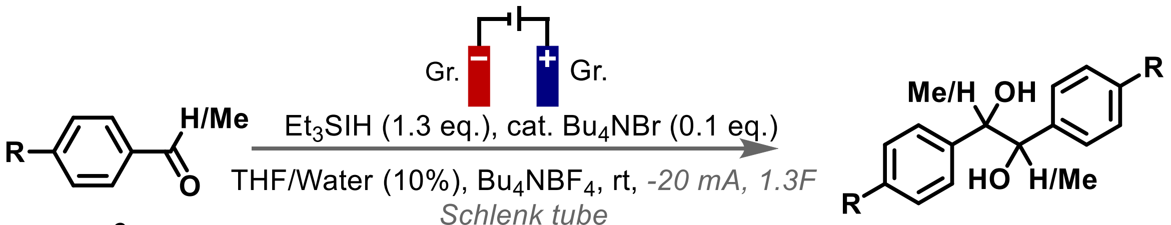

6
1 mmol

10

Under air, a 10 mL wide neck Schlenk tube equipped with a PTFE stir bar was charged with Bu_4NBF_4 (330 mg, 1.0 mmol, 1.0 eq.) Bu_4NBr (32 mg, 0.1 mmol, 0.1 eq.) and the substrate (1.00 mmol, 1.00 eq.)(if solid). THF (non-anhydrous, 4.5 mL) and water (0.5 mL) were added. Et_3SiH (0.21 mL, 1.3 mmol, 1.3 eq.) and the substrate (1.00 mmol, 1.00 eq.)(if liquid) were added via pipetting. The septum was swapped for a polypropylene stopper with two crocodile clips holding graphite plates.

Electrolysis was performed for 20 mA for 6240 s (1.3*F*). After electrolysis, the electrodes were rinsed using MeCN. The reaction mixture was then concentrated under reduced pressure, loaded onto silica gel and purified by FCC to afford the title compounds.

Cathode	Anode	Stir rate	Current	Duration	Charge
graphite plate	graphite plate	200 rpm	20 mA	6240s	1.3*F*

1,2-bis (4-methoxyphenyl) ethane-1,2-diol, 10a

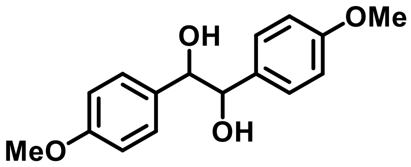

Following General Procedure 4, FCC (50-70% EtOAc in pentane) afforded an off-white solid (70%) constituting of a mixture of diastereomers (major:minor = 3:1 *nb: this ratio was found to decrease upon storage at rt)*.

Major:
^{1}H NMR (400 MHz, Chloroform-*d*) δ 7.07 – 7.02 (m, 4H), 6.79 – 6.73 (m, 4H), 4.64 (s, 2H) 3.77 (s, 6H), 2.73 (s,2H).
^{13}C NMR (101 MHz, Chloroform-*d*) δ 159.3, 132.2, 128.2, 113.6, 78.9, 55.3.

Minor:
1H NMR (400 MHz, Chloroform-d) δ 7.23 – 7.19 (m, 4H), 6.88 – 6.84 (m, 4H), 4.74 (s, 2H) 3.81 (s, 6H), 2.03 (s, 2H).
^{13}C NMR (101 MHz, Chloroform-*d*) δ 159.54, 132.12, 128.42, 113.80, 77.89, 55.35.

Data in agreement with the literature, assuming that the major isomer is *dl* and the minor isomer is *meso*.[6]

1,2-bis(4- fluorophenyl)ethane-1,2-diol, 10b

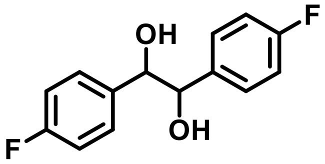

Following General Procedure 4, C_6F_6 (20.0 µL) was added for NMR yield assay at the end of the electrolysis (^{19}FNMR = quant). FCC (50-100 % EtOAc in pentane) afforded a white solid (73%) constituting of a mixture of diastereomers (major:minor = 3:1).

Major:
^{1}H NMR (400 MHz, Chloroform-*d*) δ 7.07 – 7.00 (m, 4H), 6.94 – 6.85 (m, 4H),), 4.60 (s, 2H), 3.08 (s, 2H).
^{19}F NMR (377 MHz, Chloroform-*d*) δ -114.0 (tt, *J* = 8.7, 5.4 Hz).
^{13}C NMR (101 MHz, Chloroform-*d*) δ 162.5 (d, *J* = 246.6 Hz), 135.5 (d, *J* = 3.0 Hz), 128.8 (d, *J* = 8.2 Hz), 115.2 (d, *J* = 21.5 Hz), 78.8.

Minor:
^{1}H NMR (400 MHz, Chloroform-*d*) δ 7.17 – 7.10 (m, 4H), 6.99 – 6.94 (m, 4H), 4.81 (s, 2H), 2.47 (s, 2H).
^{19}F NMR (377 MHz, Chloroform-*d*) δ -114.1 (tt, *J* = 8.7, 5.4 Hz).
^{13}C NMR (101 MHz, Chloroform-*d*) δ 162.6 (d, *J* = 246.3 Hz), 135.3 (d, *J* = 3.2 Hz), 128.8 (d, *J* = 8.2 Hz), 115.2 (d, *J* = 21.5 Hz), 77.3.

Data in agreement with the literature, assuming that the major isomer is *dl* and the minor isomer is *meso*.[6]

1,2-diphenylethane-1,2-diol, 10c

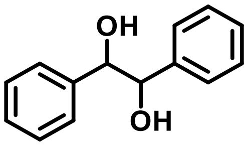

Following General Procedure 4, FCC (40-60% EtOAc in pentane) afforded a white solid (63%) constituting of a mixture of diastereomers (major:minor = 3:1).

Major:
^{1}**H NMR** (500 MHz, Chloroform-*d*) δ 7.27 – 7.22 (m, 6H), 7.16 – 7.09 (m, 4H), 4.71 (s, 2H), 3.00 (s, 2H).
^{13}**C NMR** (126 MHz, Chloroform-*d*) δ 139.9, 128.1, 127.9, 127.0, 79.1.

Minor:
^{1}**H NMR** (500 MHz, Chloroform-*d*) δ 7.35 – 7.30 (m, 6H), 7.27 – 7.22 (m, 4H), 4.84 (s, 2H),2.35 (s, 2H).
^{13}**C NMR** (126 MHz, Chloroform-*d*) δ 139.8, 128.2, 128.1, 127.1, 78.1.

Data in agreement with the literature, assuming that the major isomer is *dl* and the minor isomer is *meso*.[6]

2,3-diphenylbutane-2,3-diol, 10d

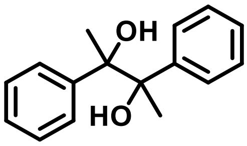

Following General Procedure 4, FCC (20-40% EtOAc in pentane) afforded a pink solid (59%) constituting of a mixture of diastereomers (major:minor = 6:1).
Major:
^{1}**H NMR** (500 MHz, Chloroform-*d*) δ 7.31 – 7.26 (m, 6H)*, 7.23 (ddd, *J* = 7.4, 3.8, 1.9 Hz, 4H)*, 2.59 (s, 2H), 1.55 (s, 6H).
^{13}**C NMR** (126 MHz, Chloroform-*d*) δ 143.4, 127.4, 127.2, 127.1, 78.9, 25.0.

Minor:
^{1}**H NMR** (500 MHz, Chloroform-*d*) δ 7.31 – 7.28 (m)*, 7.28 – 7.26 (m)*, 2.30 (s, 2H), 1.62 (s, 6H).
^{13}**C NMR** (126 MHz, Chloroform-*d*) δ 143.8, 127.3, 126.9, 126.9, 78.6, 25.1.
*overlap between Minor and Major signals.

Data in agreement with the literature, assuming that the major isomer is *dl* and the minor isomer is *meso*.[6]

2,3-Bis(4-methoxyphenyl)butane-2,3-diol, 10e

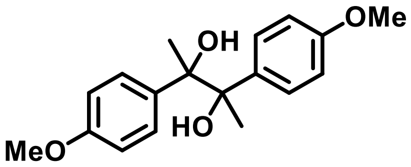

Following General Procedure 4, FCC (20-40 % EtOAc in pentane) afforded a clear oil (64%) constituting of a mixture of diastereomers (major:minor = 5:1).

Major:
^{1}H NMR (400 MHz, Chloroform-*d*) δ 7.11 – 7.04 (m, 4H), 6.80 – 6.73 (m, 4H), 3.79 (s, 6H), 2.52 (s, 2H), 1.45 (s, 6H).
^{13}C NMR (101 MHz, Chloroform-*d*) δ 158.6, 135.8, 128.6, 112.5, 78.8, 55.3, 25.1.

Minor:
^{1}H NMR (400 MHz, Chloroform-*d*) δ 7.16 – 7.11 (m, 4H), 6.76 – 6.73 (m, 4H), 3.78 (s, 6H), 2.24 (s, 2H), 1.54 (s, 6H).
^{13}C NMR (101 MHz, Chloroform-*d*) δ 158.5, 136.1, 128.2, 112.7, 78.6, 55.3, 25.3.

Data in agreement with the literature, assuming that the major isomer is *dl* and the minor isomer is *meso*.[6]

Unsuccessful reactions

Attempted sacrificial anode-free Sp^3-Sp^3 cross coupling:

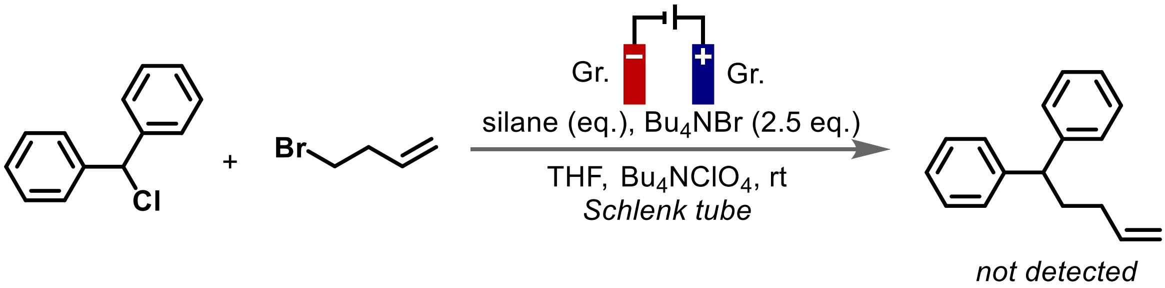

The reaction was performed with either Et_3SiH (8.0 eq.), 1,1,3,3-tetramethlydisiloxane (8.0 eq.), diphenylsilane (3.0 eq.), triethoxysilane (3.0 eq.) or dimethylphenylsilane (3.0 eq.) as the silane.

An oven-dried wide neck 10 mL Schlenk tube was taken into a nitrogen-filled glovebox and charged with Bu_4NClO_4 (200 mg, 0.6 mmol, 3.0 eq.), Bu_4NBr (260 mg, 0.8 mmol, 4.0 eq.). The tube was sealed, taken out of the glovebox, and placed under a positive pressure via a Schlenk line.

Over a flow of N_2, diphenylchloromethane (36.0 µL, 0.20 mmol, 1.00 eq.), 4-bromo-but-1-ene (61.0 µL**,** 0.60 mmol, 3.00 eq.) and the silane (3.0 or 8.0 eq.) were added via micropipette. THF (5.0 mL) was added via syringe and the mixture was sparged with N_2 for 10 s. The septum was swapped for a polypropylene stopper with two crocodile clips holding a graphite plate cathode and a graphite plate anode.

Electrolysis was performed for 30 mA for 2560 s (4*F*) *(Et_3SiH or 1,1,3,3-tetramethlydisiloxane)* or 5 mA for 11520 s (3*F*) (*diphenylsilane, triethoxysilane or dimethylphenylsilane)*. After electrolysis, the electrodes were rinsed using MeCN and the reaction mixture was concentrated under reduced pressure. After the addition of a known amount of CH_2Br_2, ^{1}HNMR analysis showed that, despite the conversion of diphenylchloromethane (<50% remaining), no significant amount of product was formed. A complex mixture was observed with multiple signals in the aromatic area (δ 6.5 – 7.5 ppm). Diphenylmethane (characteristic singlet at δ 3.9) was observed but was not the main species.

It is suspected that the silane may interact unfavourably with the formed diphenylmethyl radical.

Attempted sacrificial anode-free Birch electrochemical reduction.

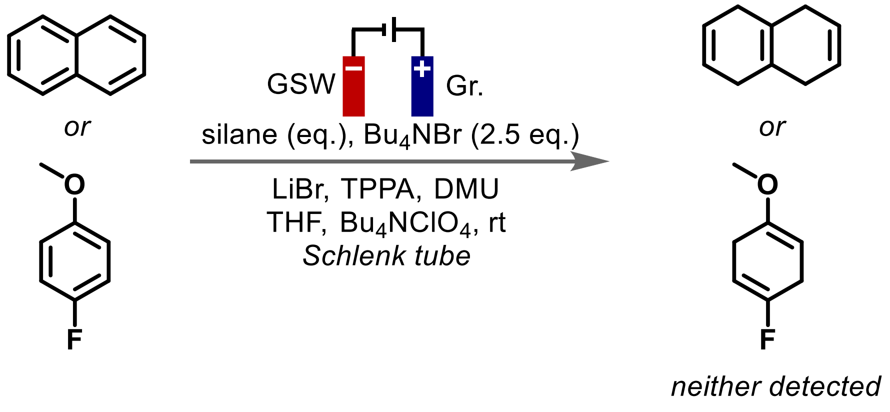

The reaction was performed with 4-methylanisole or naphthalene as a substrate, using either Et_3SiH or 1,1,3,3-tetramethyldisiloxane as a silane.

An oven-dried wide neck 10 mL Schlenk tube was taken into a nitrogen-filled glovebox and charged with LiBr (65 mg, 0.8 mmol, 8.0 eq.), *N,N*-dimethylurea (26 mg, 0.3 mmol 3.0 eq.), substrate (0.10 mmol, 1.00 eq., if solid) and Bu_4NBr (130 mg, 0.4 mmol, 4.0 eq.). The tube was sealed, taken out of the glovebox, and placed under positive pressure via a Schlenk line.

Over a flow of N_2, tris(1-pyrrolidinyl)phosphine oxide (0.23 mL, 1.0 mmol, 10 eq.), substrate (0.10 mmol, 1.00 eq., if liquid) and the silane (8.0 eq.) were added via a micropipette. THF (3.5 mL) was added via syringe. The septum was swapped for a polypropylene stopper with two crocodile clips holding a Gr. plate anode and a galvanised steel wire cathode.

Electrolysis was performed for 10 mA or 30 mA for 3-6*F*. ^{1}H NMR of the reaction mixture did not detect any product, with mostly starting material remaining.

Flow electrochemical experiments using the Electrovortex Reactor

General procedure 5 for running the ElectroVortex reactor

The chiller is turned on and set to 10 °C (IPA/MeOH, 1:1). A flask containing MeCN is attached to the inlet line and both peristaltic pumps are set to 5 $mlmin^{-1}$, the rotor is then turned to the desired rotation and the reactor is allowed to flush for 10 minutes. A flask containing the reaction solution is connected to inlet line and the inlet pump is set to the desired flow rate. The solution is then left pumping until a steady state concentration is achieved within the system, this is monitored by inline FTIR. The power supply is then turned on and set to the desired current using the constant current mode. A sample is then collected for analysis after steady state. has been observed, this takes approximately 5 reactor volumes (54 mL) of solution. After all experimentation is complete the inlet pump is opened and the reactor is flushed with solvent (MeCN) and the power supply unit is turned off. The ElectroVortex is then detached and dismantled before being cleaned with MeOH and a brush, both the inner and outer electrodes are cleaned. The vortex reactor is then reassembled and reconnected to the system and the reactor is ready for operation.

Optimisation of the electrochemical synthesis of (difluoromethyl)benzene, "Generation 1 conditions"

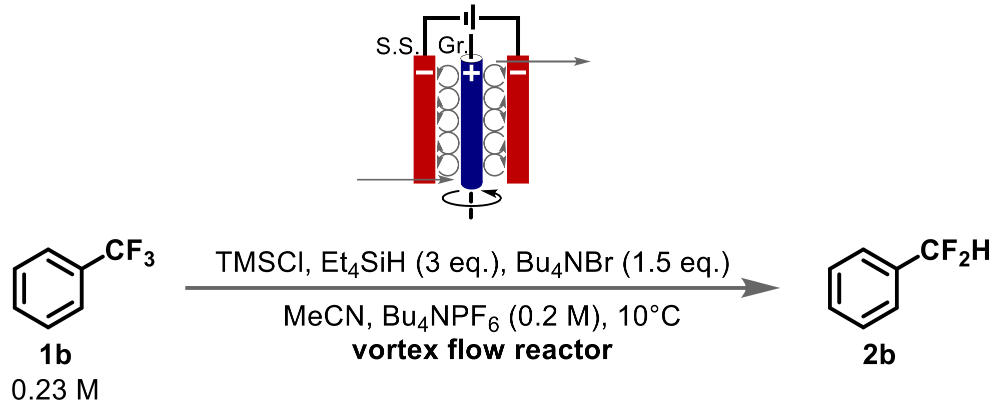

Under air, a solution of trifluorotoluene (45.0 mmol, 5.50 mL) in MeCN (150 mL) was prepared and triethylsilane (135 mmol, 21.5 mL), Bu_4NBr (67.5 mmol, 21.8 g), TMSCl (see table S9) and Bu_4NPF_6 (30.0 mmol, 11.6 g) were added. The reaction solution is then connected to inlet line **2** and the desired flow rates and rotor speed are set, general procedure **5** is then followed. Each run allows for 2 reaction conditions to be investigated, with each sample being analysed using quantitative ^{19}F NMR with fluorobenzene as an internal standard.

*Table S9: Continuous flow optimisation of the synthesis of (difluoromethyl)benzene. All yields calculated versus fluorobenzene. *Reaction performed at 0.1 M.*

Entry	TMSCl [eq.]	Current [A]	Flow Rate [mlmin^{-1}]	Rotor Speed [RPM]	*F*	Consumption [%]	Yield **2b** [%]	Yield $PhCF[H/TMS]_2$ [%]	Productivity [gday^{-1}]
1*	6.0	0.63	1.00	200	3.9	47	42	0	5.8
2	6.0	1.88	1.00	200	5.1	99	48	49	20
3	6.0	1.88	1.00	1000	5.1	93	60	36	25
4	6.0	1.88	2.00	1000	2.5	66	50	5	42
5	3.0	2.82	2.00	1000	3.8	81	64	12	54
6	3.0	3.76	2.00	1000	5.1	92	65	30	55
7	3.0	8.46	5.00	1000	4.6	96	39	48	83
8	3.0	8.56	5.00	2000	4.6	93	54	30	115
9	3.0	7.05	5.00	2000	3.8	87	68	22	143
10	3.0	8.23	5.00	2000	4.5	95	48	43	102

Multigram synthesis of (difluoromethyl)benzene in continuous flow, "Generation 1 conditions"

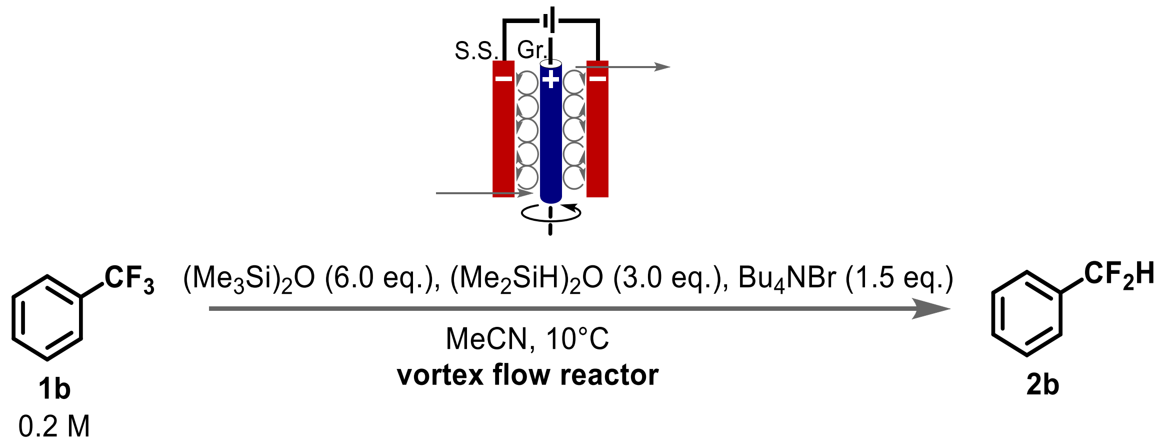

1b
0.2 M

2b

Under air, a solution of trifluorotoluene (0.36 mol, 44.2 mL) in MeCN (1200 mL) was prepared and triethylsilane (1.08 mol, 171.6 mL), Bu_4NBr (0.54 mol, 174.1 g), TMSCl (1.08 mol, 135.6 mL) and Bu_4NPF_6 (0.24 mol, 92.9 g) were added to the solution sequentially. The flow rate of the inlet pump was the set to 5 $mlmin^{-1}$ and the rotor turned to 2000 rpm, the reaction solution was then added in inlet line 2 and general procedure **5** was followed. The PSU current was set to 7.05 A (3.8*F*) and the reaction left to achieve S.S., the reaction was then left collecting in a glass bottle for 4 hours. Hourly timepoint were collected and the conversion and yield were measured by quantitative ^{19}F NMR.

Table S10: Consumption and yields over a 4h continuous flow electrolysis. All yields calculated versus fluorobenzene

Entry	Time since start of steady state [h]	Consumption [%]	Yield [%]
1	0	85	78
2	1	85	78
3	2	84	77
4	3	85	76
5	4	86	76

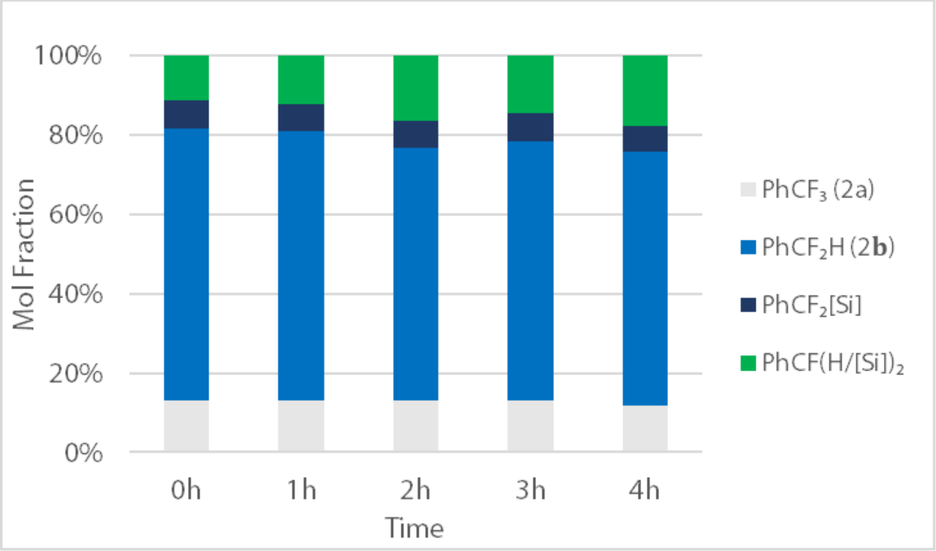

Figure S 9: Product distribution over a 4h continuous flow electrolysis, determined by quantitative ^{19}F NMR.

Late-stage functionalisation of fluoxetine (Prozac) using the electrovortex reactor, "Generation 1 conditions"

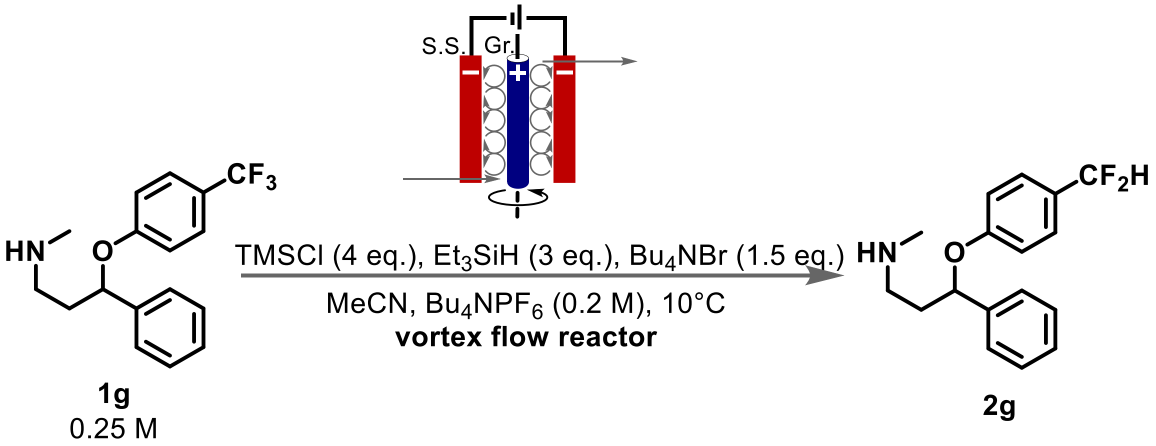

Under air, a solution of fluoxetine (30.0 mmol, 9.30 g) in MeCN (100 mL) was prepared and triethylsilane (90.0 mmol, 14.3 mL), Bu_4NBr (45.0 mmol, 14.50 g), TMSCl (120 mmol, 15.1 mL) and Bu_4NPF_6 (20.0 mmol, 7.70 g) were added to the solution sequentially. The flow rate of the inlet pump was the set to 2 mlmin^{-1} and the rotor turned to 2000 rpm, the reaction solution was then added in inlet line **2** and general procedure **5** was followed. 2 reaction conditions were investigated (Table S11), with each sample being analysed using quantitative ^{19}F NMR with fluorobenzene as an internal standard.

Table S11: Continuous flow optimisation of the synthesis of the defluorination of fluoxetine (Prozac). All yields quoted versus fluorobenzene.

Entry	Flow Rate [ml.min^{-1}]	Rotor Speed [rpm]	Current [A]	*F*	Consumption [%]	Yield **2g** [%]	Productivity [g.day^{-1}]
1	2	2000	3.8	4.7	25	25	53
2	2	2000	5.0	6.2	48	43	91

Electrochemical synthesis of (difluoromethyl)benzene, using "Generation 2 conditions"

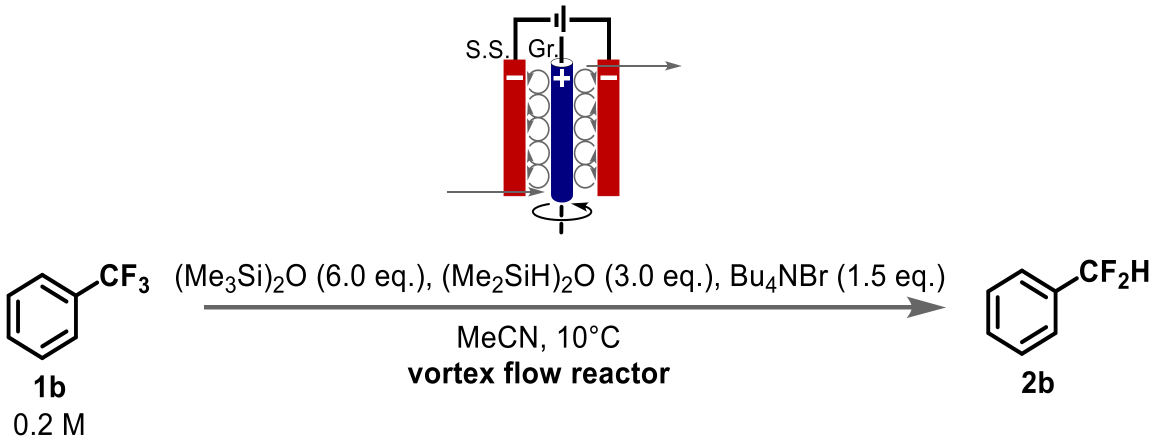

Under air, in a 100 mL volumetric flask trifluorotoluene (20 mmol, 2.45 mL) was dissolved in MeCN (50 mL). Followed by the stepwise addition of Bu_4NBr (30 mmol, 9.67 g, 1.5 eq.), tetramethyldisiloxane (60 mmol, 10.60 mL, 3.0 eq.) and hexamethyldisiloxane (120 mmol, 25.50 mL, 6 eq.). The volumetric flask was then filled up to 100 mL using MeCN and poured into a round bottom flask. As the starting solution is biphasic a magnetic stirrer and plate were added to the reaction set-up and the solution was stirred throughout the reaction. General procedure 5 was then followed, and the conditions were altered as desired (Table S12). Quantitative ^{19}F NMR analysis was used for the calculations of yields from a S.S. sample with fluorobenzene as an internal standard.

Table S12: Brief investigation of the generation 2 conditions in flow. All yields quoted versus fluorobenzene.

Entry	Flow Rate [ml.min^{-1}]	Rotor Speed [rpm]	Current [A]	*F*	Consumption [%]	Yield **2b** [%]	Yield $PhCFH_2$ [%]	Productivity [g.day^{-1}]
1	5.0	2000	6.1	3.8	91	81	10	149
2	7.5	2000	9.2	3.8	96	68	28	126

PAT analysis in continuous flow

IR Data

An inline FTIR was used to collect all data using a probe connected to a probe holder, with the reaction mixture being flowed directly through it. Spectra were obtained at 4 cm^{-1} resolution and with 16 scans. Spectra were first averaged together (4), a baseline was then applied to the obtained spectra before normalising to the MeCN peak and subtracting the solvent spectrum. An MCR model was constructed to track the concentration of reagents and product as the reaction progressed. This was done using PLS toolbox from Eigenvector and involved using calibration solutions of each reagent in a range of concentrations (0 to 1 M).

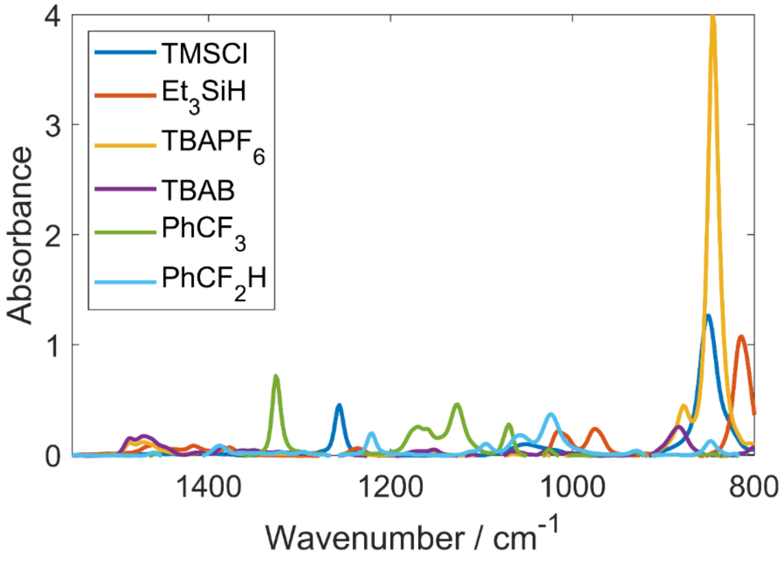

Figure S10: FTIR spectra of reagents and product in the reaction at approximately operating concentrations.

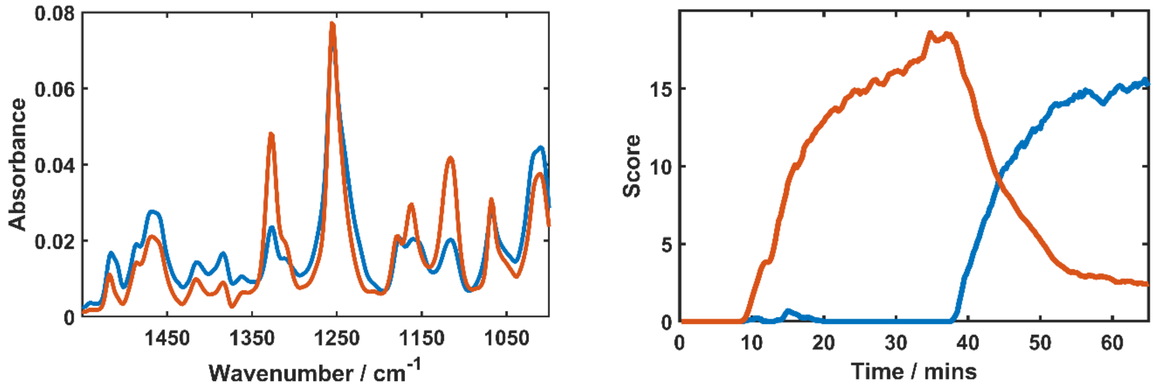

Figure S11: Left: MCR spectral loads showing starting reagents (orange) and product (blue). Right: MCR scores of each spectral load showing the reagents flow in and then converted to products when the current is turned on. This provides a qualitative indication how the reaction progresses.

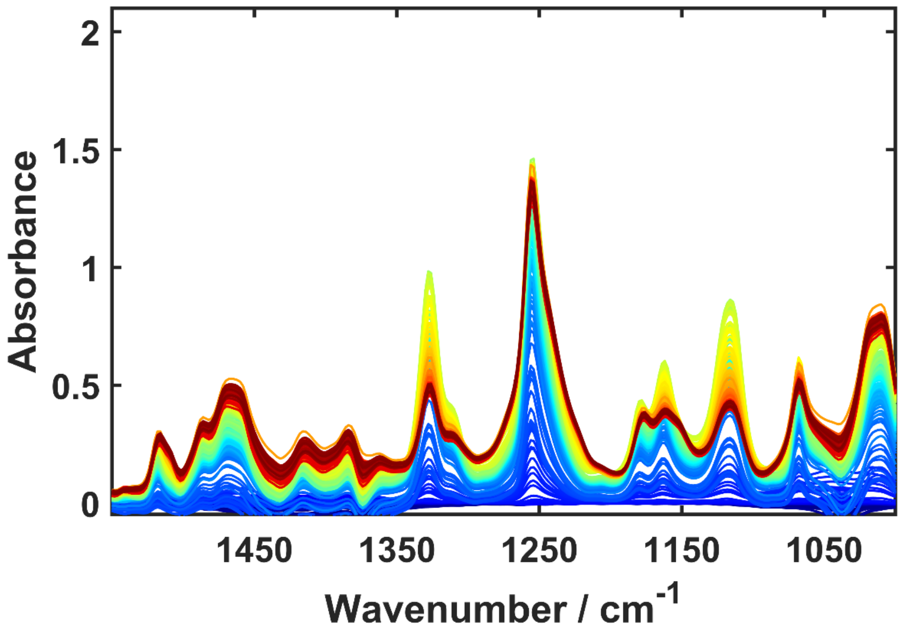

Figure S12: FTIR spectra of the hydrodefluorination of Prozac in continuous flow. Blue is the starting materials flowing in, and green to yellow is the consumption of Prozac as the current is applied.

References

(1) Box, J. R.; Atkins, A. P.; Lennox, A. J. J. Direct Electrochemical Hydrodefluorination of Trifluoromethylketones Enabled by Non-Protic Conditions. *Chem. Sci.* **2021**, *12* (30), 10252–10258.

(2) Box, J. R.; Avanthay, M. E.; Poole, D. L.; Lennox, A. J. J. Electronically Ambivalent Hydrodefluorination of Aryl-CF3 Groups Enabled by Electrochemical Deep-Reduction on a Ni Cathode. *Angew. Chem. Int. Ed.* **2023**, *62* (12). https://doi.org/10.1002/anie.202218195.

(3) Kise, N.; Agui, S.; Morimoto, S.; Ueda, N. Electroreductive Acylation of Aromatic Ketones with Acylimidazoles. *J. Org. Chem.* **2005**, *70* (23), 9407–9410.

(4) Oi, S.; Moro, M.; Fukuhara, H.; Kawanishi, T.; Inoue, Y. Rhodium-Catalyzed Addition of Arylstannanes to Carbon–Heteroatom Double Bond. *Tetrahedron* **2003**, *59* (24), 4351–4361.

(5) Lu, L.; Siu, J. C.; Lai, Y.; Lin, S. An Electroreductive Approach to Radical Silylation via the Activation of Strong Si–Cl Bond. *J. Am. Chem. Soc.* **2020**, *142* (51), 21272–21278.

(6) Wang, Q.; Shonhe, C.; Ji, S.-H.; Wu, J.; Zhou, J.; Cai, Y.-R. Discovery of Anodic Thiourea Oxidation as a Sustainable Counter Reaction to Boost Electro-Reductive Organic Transformations. *ACS Sustain. Chem. Eng.* **2023**, *11* (6), 2449–2454.
